# IFNγ inhibits G-CSF induced neutrophil expansion and invasion of the CNS to prevent viral encephalitis

**DOI:** 10.1371/journal.ppat.1006822

**Published:** 2018-01-19

**Authors:** Chandran Ramakrishna, Edouard M. Cantin

**Affiliations:** Department of Molecular Immunology, Beckman Research Institute of City of Hope, Duarte, California, United States of America; Yale University School of Medicine, UNITED STATES

## Abstract

Emergency hematopoiesis facilitates the rapid expansion of inflammatory immune cells in response to infections by pathogens, a process that must be carefully regulated to prevent potentially life threatening inflammatory responses. Here, we describe a novel regulatory role for the cytokine IFNγ that is critical for preventing fatal encephalitis after viral infection. HSV1 encephalitis (HSE) is triggered by the invasion of the brainstem by inflammatory monocytes and neutrophils. In mice lacking IFNγ (GKO), we observed unrestrained increases in G-CSF levels but not in GM-CSF or IL-17. This resulted in uncontrolled expansion and infiltration of apoptosis-resistant, degranulating neutrophils into the brainstem, causing fatal HSE in GKO but not WT mice. Excessive G-CSF in GKO mice also induced granulocyte derived suppressor cells, which inhibited T-cell proliferation and function, including production of the anti-inflammatory cytokine IL-10. Unexpectedly, we found that IFNγ suppressed G-CSF signaling by increasing SOCS3 expression in neutrophils, resulting in apoptosis. Depletion of G-CSF, but not GM-CSF, in GKO mice induced neutrophil apoptosis and reinstated IL-10 secretion by T cells, which restored their ability to limit innate inflammatory responses resulting in protection from HSE. Our studies reveals a novel, complex interplay among IFNγ, G-CSF and IL-10, which highlights the opposing roles of G-CSF and IFNγ in regulation of innate inflammatory responses in a murine viral encephalitis model and reveals G-CSF as a potential therapeutic target. Thus, the antagonistic G-CSF-IFNγ interactions emerge as a key regulatory node in control of CNS inflammatory responses to virus infection.

## Author summary

Successful resolution of infections requires a balanced immune response that recognizes and destroys invading pathogens, without provoking excessive damage to the host. Interferon gamma (IFNγ) is an important pleiotropic cytokine, which regulates host immunity at key stages of the immune response. However, much less is known about its regulatory activities during inflammation, especially in viral infections. Here, we show a novel role for IFNγ in regulating innate immune responses during a viral infection. HSV1 induced encephalitis is triggered by inflammatory monocytes and neutrophils invading the brainstem. In the absence of IFNγ, G-CSF and IL-6 levels but not IL-17 and GM-CSF, were highly elevated. This resulted in uncontrolled expansion and infiltration of pathogenic neutrophils into the CNS causing fatal encephalitis in IFNγ deficient (GKO) but not wildtype mice. Depletion of G-CSF in GKO mice abolished neutrophil expansion, reinstated IL-10 secretion by T cells, resulting in protection from encephalitis. Our study reveals a novel G-CSF regulated pathway, independent of GM-CSF and IL-17, that controls neutrophil responses to viral infection. Importantly, IFNγ emerges as a key regulator of the inflammatory response to virus infection and this functionality is distinct from its direct anti-microbial activities.

## Introduction

Early detection and eradication of invading pathogens by the immune response, without excessive bystander damage to the inflamed organ, is the most desirable outcome for the host [1]. Efficient pathogen control requires activation of innate immune cells, including BM derived neutrophils and monocytes, and their recruitment to the infected tissue to initiate pathogen clearance and shape the ensuing immune response [2–4]. Although the recruitment of inflammatory cells to the target organ is directed by the establishment of a chemokine gradient [5], the generation of inflammatory cells is facilitated by a hematopoietic response program, termed emergency hematopoiesis, which is characterized by greatly increased de novo neutrophil and monocyte production from BM progenitor cells [6–9]. Under steady state conditions, hematopoiesis is a tightly regulated process; but, to satisfy the enormous demand for leukocytes after infection, a complete overhaul of the program occurs. This overhaul results in emergency hematopoiesis and the subsequent production of substantial numbers of the desired cell type(s) [9]. However, under some conditions, impaired regulation of emergency hematopoiesis results in unrestrained inflammation and toxicity to the host [2, 10–14]. Several intrinsic and extrinsic factors influence emergency hematopoiesis [7, 15–21]. In the last decade, various reports have elucidated the key role played by cytokines in the regulation of both steady state and emergency hematopoiesis [7, 15–17].

Cytokines are master regulators of the immune response, and they are produced during immunological stress, such as infections [15, 22]. The evolution of an immune response can be predicated by the amount and location of cytokine production, cytokine access to other cells, and the stage of infection during which the cytokine is produced. Several cytokines, including IL-6, TNF-α, GM-CSF, G-CSF, IL-1α, and the IFNs, control leukocyte production and emigration during steady state and emergency hematopoiesis [7, 8, 17, 23, 24]. G-CSF and GM-CSF influence myeloid homeostasis and GM-CSF is thought to be a key mediator of Th17-driven neutrophil-mediated inflammatory diseases [25–28]. Some cytokines, such as IFN, play prominent dual roles in both development and homeostasis of the immune system and immunity to pathogens [23, 24, 29–31]. Type I IFN subtypes (α/β) are considered indispensable for antiviral protection while IFNγ, a type II IFN, is essential for bacterial eradication and also for controlling replication of some viruses [31–34]. Because we found increased IFNγ, rather than IFNα/β, following HSV infection in mice, we investigated the role of IFNγ in HSV-induced inflammation of the CNS.

IFNγ has long been considered a prototypical pro-inflammatory Th1 cytokine that drives cell-mediated immunity. However, its role in inflammation remains controversial, even though evidence for its immuno-regulatory role has gradually accumulated over the last decade [30, 35–37]. Several organ-specific autoimmune and chronic inflammatory diseases have been associated with dysregulated Th1 responses, but unexpectedly, these diseases progress at accelerated rates in mice deficient in IFNγ or its receptor due to increased Th17 responses [30, 36–40]. These observations have challenged the dogmatic pro-inflammatory role of IFNγ, as well as the Th1-Th2-Th17 paradigm, as a rationale for explaining autoimmune disorders. We reported that mortality from viral encephalitis is primarily linked to exaggerated innate inflammatory monocyte and neutrophil responses, rather than viral replication induced tissue damage [41, 42]. In the current report, we establish a novel role for IFNγ in the development and resolution of protective CNS innate immune responses, after infection with HSV, a neurotropic 〈-herpesvirus. In the absence of IFNγ, production of G-CSF, but not GM-CSF or IL-17, was greatly increased resulting in the generation of apoptosis-resistant, spontaneously degranulating neutrophils. These neutrophils invaded the CNS in large numbers, causing fatal HSV-induced encephalitis (HSE). This increase in G-CSF and neutrophils suppressed T-cell functionality and importantly, ablation of G-CSF abolished neutrophilia, restored T cell functionality and protected mice from fatal HSE. Thus, our study reveals a novel regulatory role for IFNγ in the control of G-CSF induced neutrophilia and further illuminates its role in protection from viral encephalitis.

## Results

### IFNγ and IL-10 are the predominant cytokines secreted following HSV infection

**Fig 1:**
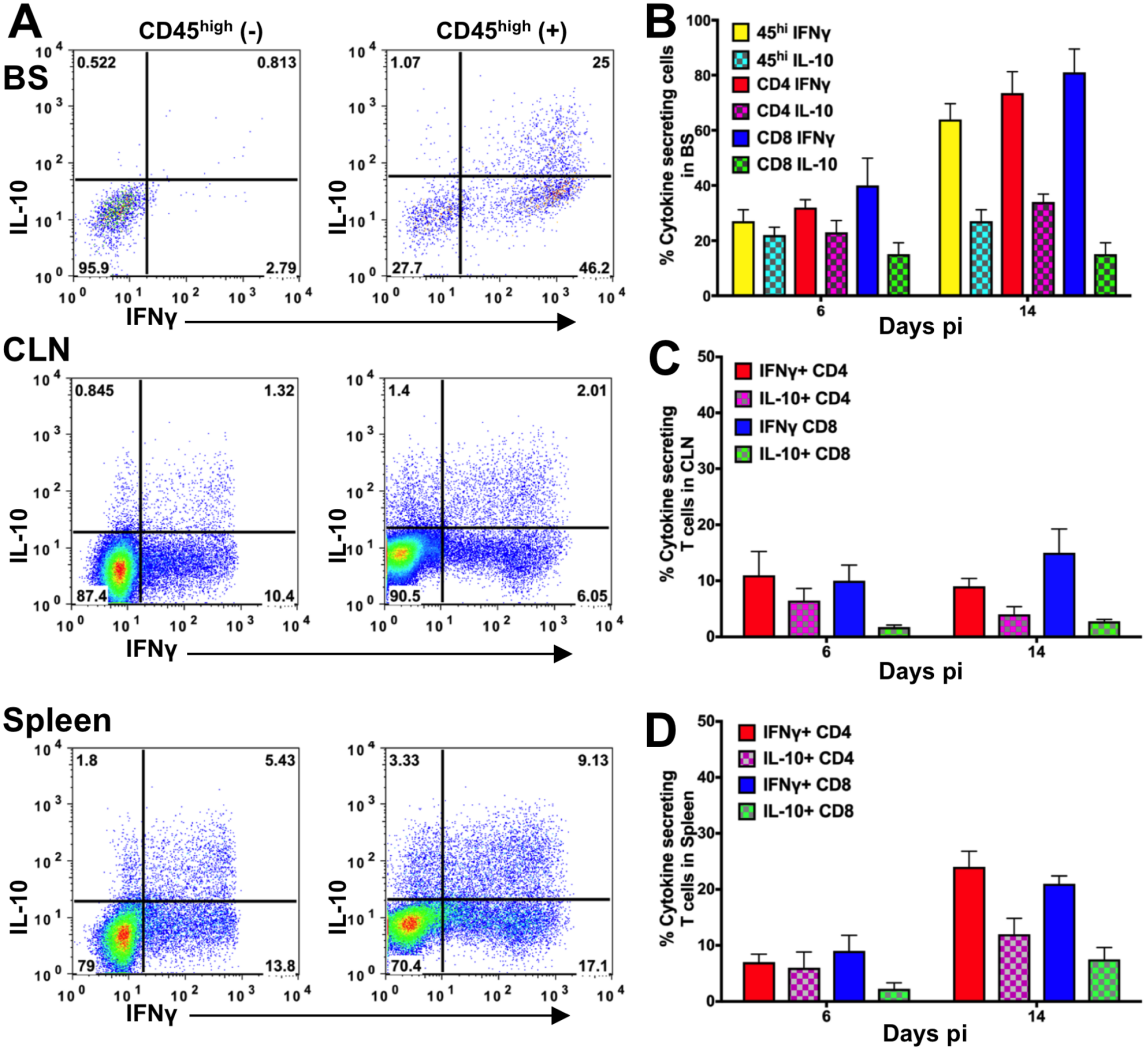
IFNγ and IL-10 dominate the cytokine profile of T cells isolated from HSV-infected WT mice. **(A)** Mononuclear cells isolated from brain stem (BS, top), cervical lymph nodes (CLN, middle) or spleen (bottom) on day 6 or 14 pi from HSV-infected WT mice were probed for IL-10 and IFNγ by intracellular cytokine staining (ICS). Representative FACS plots show cells isolated at day 14 pi. All flow cytometry plots show antigen-stimulated cells (+) except for top left panel “for BS (-)”. Bar plots depict % IL-10- or IFNγ-secreting CD45^high^, CD4 or CD8 T cells in the **(B)** BS, **(C)** CLN or **(D)** spleen at day 6 and 14 pi. Data representative of 2 (day 14 pi) -3 (day 6 pi) experiments are shown as mean +/- SD (n = 3–4 mice / time point).

We showed previously that IL-10-deficient and T- and B cell-deficient Rag^-/-^ mice succumb to fatal HSE resulting from excessive infiltration by inflammatory Ly6C^high^ monocytes (IMs) into the brainstem (BS) [42, 43]. To determine the mechanism(s) by which inflammation, specifically IM infiltration, is suppressed by T cells in WT mice, we measured cytokine production by T cells isolated from WT mice at days 6 and 14 post-infection (pi). We determined the T-cell cytokine levels by intracellular cytokine staining (ICS) using flow cytometry, and serum cytokine levels by enzyme-linked immunosorbent assay (ELISA). IL-10 and IFNγ were the major cytokines produced by T cells in the BS, draining cervical lymph nodes (CLN) and spleen (Fig 1A–1D). At day 6 pi in the BS, we observed 20–40% IL-10^+^ and IFNγ^+^ T cells, which almost doubled by day 14 pi (Fig 1B). Notably, most (> 80%) of the cytokine-secreting CD4 and CD8 T cells in the BS were IFNγ^+^ at day 14 pi. Although not as impressive as in the BS, IFNγ^+^ and IL-10^+^ T cells comprised the predominant cytokine-producing subsets detected in the CLN and spleen (Fig 1C and 1D). The cytokines and chemokines that were analyzed in the sera and BS of infected WT mice at day 6 pi are listed in S1 and S2 Figs respectively and these were either undetectable or altered relative to day 0 levels. Not surprisingly, the IFNγ-driven chemokines, such as IP-10 (CXCL10) and MIG (CXCL9), dominated the chemokine profile, concordant with our prior observation that T cells are the predominant cell population in the BS infiltrate of WT mice [42]. Since, the major cytokines detected in serum are IL-27, IL-13, IFNγ, IL-10 and IL-18 (S1 Fig), we conclude that the infected WT mice express a balanced Th1/Th2 signature. Previously, we found an anti-inflammatory role for IL-10, which suppressed the generation of IM, thereby preventing HSE in WT but not IL-10 knockout (IL-10KO) mice [42]. We now show that IFNγ, a classical pro-inflammatory cytokine, is present at substantially greater levels than the anti-inflammatory cytokine IL-10 in HSV-infected WT mice.

### T cells but not IFNγ are required for control of virus replication

**Fig 2:**
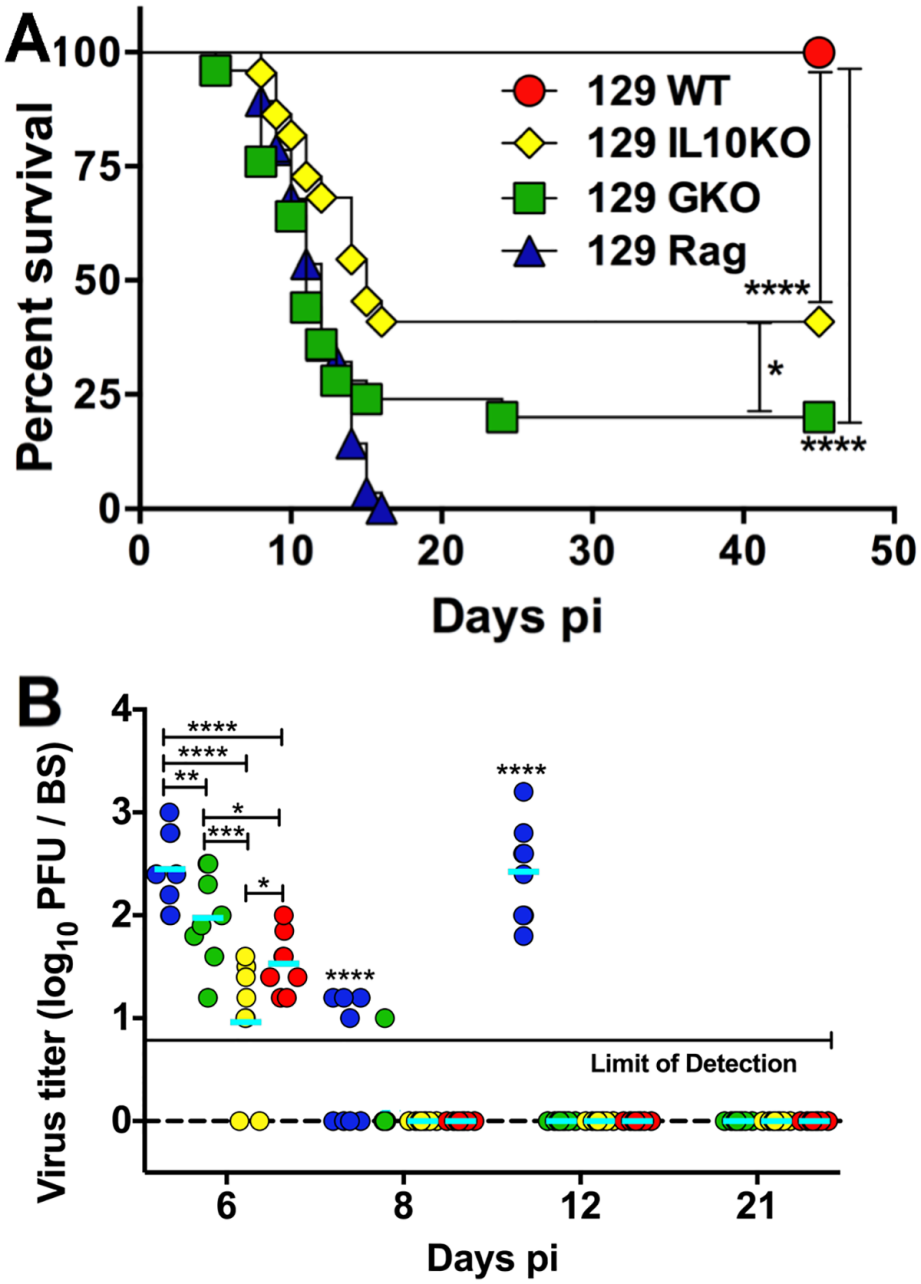
IFNγ is required for survival but not control of HSV replication. **(A)** HSV-infected WT, IL-10KO, GKO and Rag^-/-^ mice were monitored for survival from HSE; data are representative of 3–5 experiments (n = WT: 40, IL-10KO: 22, GKO: 36, Rag^-/-^: 28 mice). ****p<0.0001; *p<0.05. **(B)** BS was isolated at the indicated time points from HSV-infected WT, IL-10KO, GKO or Rag^-/-^ mice and virus titers determined by plaque assay (n = 8–10 mice per time point), *p<0.05, **p<0.01, ***p<0.001, ****p<0.0001. p<0.0001 for all time points for Rag^-/-^ mice compared to WT and IL-10KO mice. All Rag^-/-^ mice had died by day 16 pi.

To investigate the role of IFNγ in HSE, we infected WT, IFNγ KO (GKO), IL-10KO, and Rag^-/-^ mice and monitored their survival. Unexpectedly, 80% of the GKO mice succumbed to infection within 14 days **(**Fig 2A), in contrast to the WT mice, which all survived. As previously reported, 60% of the IL-10KO mice and all of the Rag^-/-^ mice succumbed to HSV infection (Fig 2A) [42, 43]. Since Rag^-/-^ mice lack T and B cells and GKO and IL-10KO mice lack IFNγ and IL-10 respectively, these results indicate that IFNγ and IL-10 secreted by infiltrating T cells are required for optimal protection from HSE mortality [42, 43].

To determine if GKO mice succumbed due to uncontrolled virus replication, we measured the levels of infectious virus in the trigeminal ganglion (Tg) and BS tissues from GKO mice. HSV1 was undetectable after day 8 pi in the Tg and BS of GKO mice, similar to WT and IL-10KO mice (Fig 2B and S3A Fig) and most of the GKO mice died after day 8 pi, which discounts virus-induced cytopathology as the cause of mortality. As expected, virus replication was not controlled in Rag^-/-^ mice (Fig 2B). These results confirm that T cells control acute HSV replication via an IFNγ independent mechanism, consistent with prior studies in mice and humans [32, 33, 43–45].

### Unrestrained CNS inflammation in the absence of IFNγ

**Fig 3:**
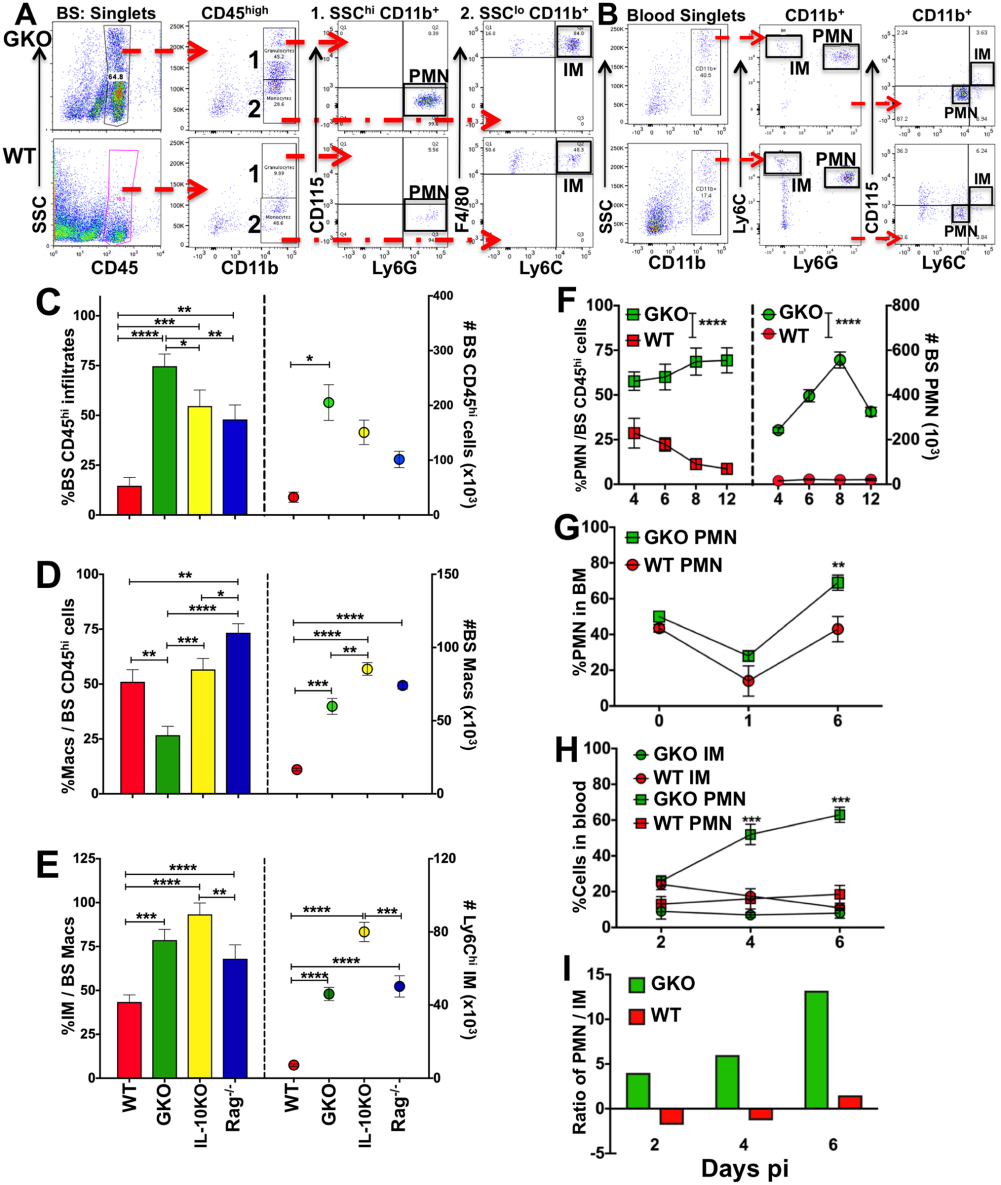
Neutrophil output from BM and CNS invasion is augmented in the absence of IFNγ. Representative flow cytometry plots depicting the gating strategy used to distinguish neutrophils (PMN) and inflammatory monocytes (IM) in the **(A)** BS and **(B)** blood of HSV-infected GKO (top row) and WT (bottom row) mice: **(A)** BS mononuclear cells isolated at day 4 pi show CD45^high^ infiltrating leukocytes, CD45^high^ SSC^high^ CD11b^+^ CD115^-^ Ly6G^high^ neutrophils (1. PMN) and F480^+^ CD115^+^ Ly6C^high^ monocytes (2. IMs); and **(B)** blood mononuclear cells at day 6 pi show SSC^high^ CD11b^+^ CD115^-^ Ly6G^+^ Ly6C^int^ PMN and CD115^+^ Ly6G^-^ Ly6C^high^ IMs. Bar plots depicting **(C)** % (left y-axis) and # (right y-axis) of CD45^high^ infiltrating cells in the BS of WT, IL-10KO, GKO and Rag^-/-^ mice at day 6 pi, **(D)** % (left y-axis) and # (right y-axis) of CD115^+^ F480^+/lo^ monocytes / macrophages within the BS CD45^high^ infiltrates, **(E)** % (left y-axis) and # (right y-axis) of Ly6C^high^ IMs within the BS CD45^high^ F480^+^ monocytes / macrophages. Line plots showing **(F)** % (left y-axis) and # (right y-axis) of Ly6G^+^ PMN within BS CD45^high^ infiltrates in GKO and WT mice at the indicated time points, **(G)** % CD11b^+^ Ly6G^+^ neutrophils within BM (1 leg = femur + tibia) at the indicated time points, **(H)** % CD11b^+^ Ly6G^+^ PMN or Ly6C^high^ IMs in the blood of GKO and WT mice, and **(I)** the ratio of PMN to IM in the blood of GKO and WT mice at the indicated time points. Data representative of 2–3 experiments are shown as mean +/- SD; *p<0.05, **p<0.01, ***p<0.001, ****p<0.0001.

Excessive CNS infiltration by the pathogenic CD11b^+^ Ly6C^high^ IM subset, which is characteristic of fatal HSE, is limited by the anti-inflammatory cytokine IL-10 [41, 42]. Because IFNγ plays an important role in myeloid responses, we investigated whether GKO mice succumbed to HSE due to inefficient control of BS inflammation, specifically IM infiltration. As early as day 4 pi, CD45^high^ infiltrates composed 65% of the mononuclear cell population within the BS of GKO mice and remained the dominant population in the BS of GKO mice at day 6 pi, (Fig 3A and 3C). Notably these cells were also present at high levels in the brain and spinal cord of GKO but not WT mice (S3B Fig). Relative CD45^high^ infiltrate levels in the BS were ranked in the order: GKO > IL-10KO > Rag^-/-^ >>> WT mice (Fig 3C), which implies surprisingly that suppression of CNS infiltration requires T cells secreting both IFNγ and IL-10. We next determined if, similar to IL-10KO mice, IMs were the major CD45^high^ infiltrating subset in the BS of the GKO mice [42]. As anticipated, most of the monocytes / macrophages (CD45^high^ SSC^low^ CD11b^+^ CD115^+^ Ly6G^-^ F480^+/lo^) in the BS of GKO, IL-10KO and Rag^-/-^ mice, but not WT mice, expressed high levels of Ly6C, characteristic of the IM subset (Fig 3A, 3D and 3E) [42]. However, infiltration of monocytes / macrophages into the GKO BS was significantly reduced compared to WT, IL-10KO and Rag^-/-^ mice (Fig 3D). Interestingly, although macrophages only accounted for a minor fraction (25%) of the total CD45^high^ infiltrate in the GKO BS, conversion to numbers revealed a three fold increase in macrophage numbers in the BS of GKO mice compared to WT mice (Fig 3D). These data suggest that an alternate CD45^high^ cell subset, possibly neutrophils, dominates BS infiltrates in GKO mice. In prior studies, we could not determine the specific role of neutrophils in HSE due to cross reactivity of the neutrophil specific antibodies available at that time [41].

### IFNγ controls neutrophil expansion and invasion of the BS

We next determined whether neutrophils (PMN) dominated the BS infiltrates in GKO mice. Neutrophils (CD45^high^ SSC^high^ CD11b^+^ Ly6G^+^ CD115^-^) comprised ~60–75% of the CD45^high^ infiltrates in the BS of GKO mice at days 4–12 pi, but were reduced in the BS of WT mice at days 4–6 pi (~30%) and diminished after day 6 pi (Fig 3A and 3F). Amazingly by day 8 pi when PMNs were on the decline in the BS of WT mice, PMN numbers were increased 30 fold in the GKO BS compared to the WT BS (Fig 3F). Furthermore, neutrophils isolated from either the BM or blood of GKO mice were increased compared to WT mice (Fig 3B, 3G and 3H). Thus, in the absence of IFNγ, neutrophils were the predominant BM-derived population in the BS of GKO mice. Importantly, the ratio of PMNs to IMs in the blood of GKO mice was much higher than in WT mice, and increased from day 2 to 6 pi (Fig 3B and 3I). Because IL-10 is important for inhibiting neutrophil responses [15, 46], we analyzed the ratio of neutrophils to IMs in the blood of IL-10KO and Rag^-/-^ mice. The ratio was reversed for IL-10KO but not Rag^-/-^ mice at days 4 and 6 pi, implying that control of IM, but not neutrophil expansion, requires IL-10 (S3C Fig). Furthermore, IL-10KO T cells secreted substantial amounts of IFNγ, similar to WT T cells further emphasizing IFNγ’s role in neutrophil contraction (S3D Fig and Fig 1B). These data suggest that during viral infection, IFNγ rather than IL-10 is the major regulator of neutrophil output, especially at later time points after infection.

### Regulatory CD4 T cells do not prevent HSE in the absence of IFNγ

**Fig 4:**
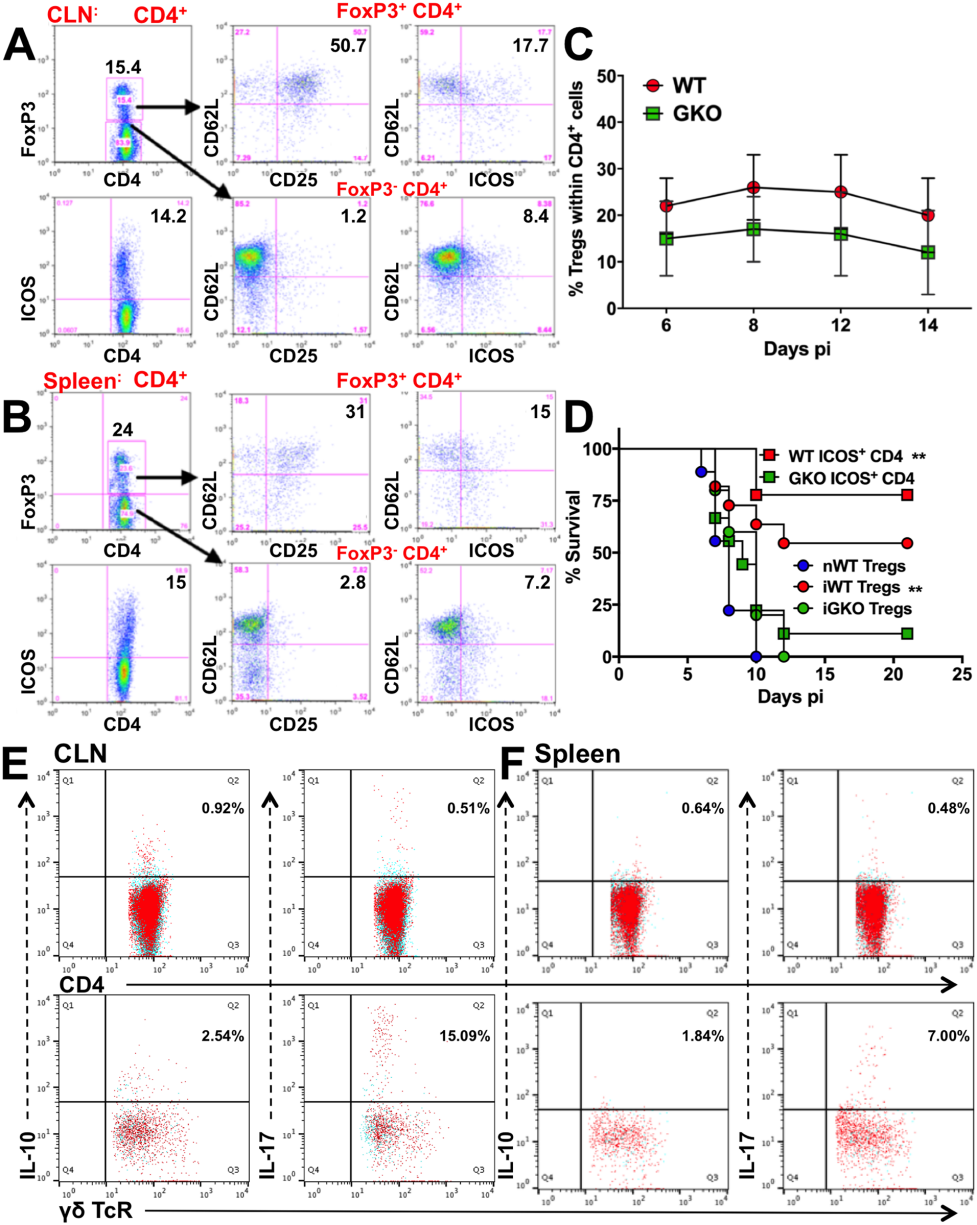
Regulatory CD4 T cells are impaired in the absence of IFNγ. FoxP3^+^ (top) and ICOS^+^ (bottom) CD4 T cells (left) in **(A)** CLN and **(B)** spleen of WT mice at day 8 pi. FoxP3^+^ (top row) and FoxP3^-^ (bottom row) CD4 T cells probed for CD62L and CD25 expression (middle) and ICOS expression (right). **(C)** % FoxP3 Tregs within splenic CD4 T cells isolated from WT or GKO mice at the indicated time points; data representative of 2–3 experiments are shown as mean ± SD. **(D)** FoxP3^+^ CD25^+^ CD4 T cells (Tregs, 5x10^6^) or ICOS^+^ (10^7^) CD4 T cells isolated from naïve WT (nWT), infected WT (iWT) or GKO (iGKO) mice at day 8 pi were adoptively transferred to naïve WT recipients, which were then challenged with HSV but did not receive IVIG, and monitored for survival (n = 6–8 mice). **p = 0.002. CD4 (top row) or γδ (bottom row) T cells isolated from **(E)** CLN or **(F)** spleen of GKO mice at day 6 pi were probed for IL-10 and IL-17 by ICS. Blue dots indicate no antigenic stimulation; red dots indicate cells stimulated with PMA + ionomycin + heat-killed HSV (HK-HSV).

Since FoxP3^+^ Tregs and ICOS^+^ CD4 T cells have been shown to moderate inflammation, we examined GKO and WT mice for regulatory CD4 T cells during HSE. IL-10-secreting FoxP3^+^ Tregs and ICOS^+^ FoxP3^-^ CD4 Tr1 cells, previously shown to protect against HSV challenge, were expanded in the CLNs and spleens of WT mice (Fig 4A and 4B) [42]. We have shown previously that Tregs were induced in peripheral lymphoid organs of WT mice but were not detected in the BS [42]. To determine if these cells were induced in GKO mice, we analyzed CLN, spleen, and BS for FoxP3 and ICOS expression on CD4 T cells (Fig 4C, S4A and S4E Fig). Unexpectedly, Tregs were reduced in the spleens of GKO mice compared to WT mice and absent in the BS of GKO mice (Fig 4C and S4E Fig). Also, adoptive transfer of FoxP3^+^ CD25^+^ CD4 Tregs or ICOS^+^ CD4 T cells from infected WT, but not GKO or naïve WT mice protected naïve WT recipients from HSE, which suggests the GKO Tregs were unable to suppress inflammatory innate immune responses (Fig 4D); we have shown previously that IL-10 secreting Tregs prevented HSE by suppressing innate inflammatory responses [42]. Intriguingly, the IL-10^+^ CD4 T cells that expanded in WT mice (Fig 1) [42] were not detected in the BS, CLN or spleen of GKO mice (Fig 4E and 4F: top row, S4B–S4D Fig), consistent with their inability to protect from HSE (Fig 4D). Similarly, IL-4^+^ and TNF^+^ CD4 T cells were also absent in spleen (S4B Fig). IFNγ, the protypical Th1 cytokine, is known to suppress Th2 cells and Tregs, but our data suggest paradoxically that during HSV infection IFNγ may be responsible for the fitness and function of regulatory T cells, revealing the complexity of IFNγ’s regulatory mechanisms [15, 35].

**Fig 5:**
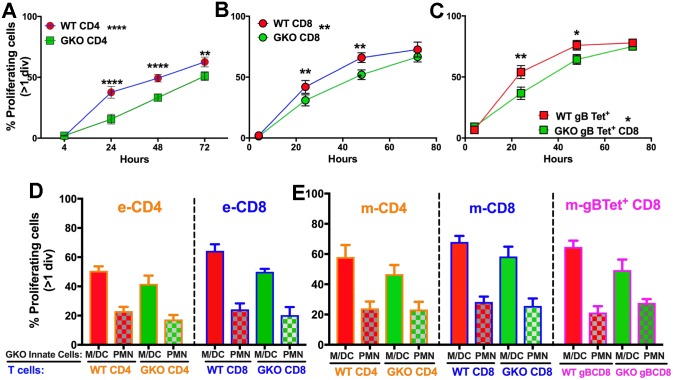
GKO Neutrophils have a GDSC phenotype and suppress T cell proliferation. **(A-C)**, Spleen cells isolated at day 6 pi from HSV infected WT or GKO mice were labeled with CFSE and stimulated with HK-HSV to determine effector (e) T cell proliferation at indicated times post culture. GKO or WT T cells that have divided at least one time are depicted as line plots as % proliferating **(A)** eCD4, **(B)** eCD8 T or (**C**) H-2K^b^ HSV-1 specific gB_498_ e-tetramer^+^ (e-Tet^+^) CD8 T cells. Linear regression analysis showed significant deviation of slope for GKO CD4 (P = 0.041), GKO CD8 (P = 0.0232) and GKO gB_498_ tet^+^ CD8 (P = 0.0171) but not WT T cells. **(D-E)** GKO Innate cells including PMN (CD11b^+^ Ly6G^+^) or M/DC (Ly6G^-^ CD11b^+^ / CD11c^+^ cells) isolated from blood of GKO mice at day 6 pi cultured with CFSE labeled GKO or WT T cells and CD19^+^ B cells isolated from spleen cells as in **A-C** were assessed for suppression of proliferation of **(D)** eCD4 and eCD8 T cells and **(E)** memory (m) CD4 and mCD8 T cells and m-H-2K^b^ HSV-1 gB_498-505_ tetramer^+^ (m-Tet^+^) CD8 T cells. Effector (e) T cells were obtained from spleens of HSV infected WT or GKO mice at day 6 pi while memory (m) T cells were obtained from spleens of immunized mice at day 21 pi. Data is combined from 2 experiments, n = 6 mice per group. PMN: Neutrophils, M/DC: Monocytes/Dendritic cells.

We next asked whether suppression of effector T-cell function during acute infection of GKO mice results from induction of myeloid- or granulocyte-derived suppressor cells (MDSCs / GDSCs) [47]. Spleen cells isolated at day 6 pi from GKO mice (containing high numbers of neutrophils) or WT mice (containing few neutrophils) were labeled with CFSE, then incubated with heat-killed HSV (HK-HSV) and analyzed at various time points for dilution of CFSE. Although neither WT nor GKO T cells proliferated after 4 h of culture (S5A Fig), by 24 h WT CD4 and virus-specific CD8 T cells proliferated more rapidly than their GKO counterparts (Fig 5A–5C). However, after 48 h of culture, proliferation rates were similar for both WT and GKO T cells (Fig 5A–5C), suggesting that the suppressive activity of GKO neutrophils declines over time, likely due to their rapid death in culture. Furthermore, in vitro WT effector CD4 and CD8 T cell proliferation was suppressed in the presence of GKO CD11b^+^ Ly6G^+^ PMN but not CD11b^+^ Ly6G^-^ monocytes or CD11c^+^ DCs (M / DCs) that were isolated at day 6 pi from blood of HSV infected GKO mice, confirming that suppression was mediated by GKO GDSCs and not other suppressor cells including Tregs (Fig 5D). To determine if similar suppressive effects were observed for memory T cells, CD11b^+^ Ly6G^+^ neutrophils or M / DCs isolated from the blood of infected GKO mice at day 6 pi were incubated with CFSE-labeled spleen cells isolated from HSV immunized WT mice at day 21 pi and memory T-cell proliferation was analyzed in vitro. Indeed, WT memory CD4, CD8 and virus specific gB_498-505_ tetramer^+^ (Tet^+^) CD8 T cells reactive to virus antigens (HK-HSV) proliferated at reduced rates (~10%) when cultured with GKO neutrophils, compared to m / DCs (Fig 5E). Although, WT memory CD4 and CD8 T cells proliferation was reduced in the presence of GKO PMNs compared to WT PMNs presenting HK-HSV antigen (S5B and S5C Fig), virus-specific WT and GKO CD8 T cells cultured with the immunodominant H-2K^b^ HSV gB_498-505_ peptide proliferated at similar rates (S5D Fig), suggesting that the suppressive effects of GKO GDSCs depends on the cytosolic proteolysis of viral antigens but can be overcome with an optimal concentration of immunodominant peptide. To determine if suppression by GDSC can also be overcome by stimulation with αCD3 and αCD28, CFSE-labeled splenocytes containing memory CD4 or CD8 T cells isolated from HSV-infected WT or GKO mice at day 25 pi were incubated with soluble αCD3 and αCD28 in the presence of Ly6G^+^ neutrophils isolated from blood of infected GKO mice at day 6 pi. Similar rates of proliferation were observed for both WT and GKO memory T cells (S5E and S5F Fig). Additionally, very few exhausted CD4 and CD8 T cells were observed in the two groups, as <5% of cells expressed high levels of PD-1 (S5B–S5D Fig). Overall, these data suggest that the suppressive effects of GDSCs are specific to virus antigens that require cytolytic processing but could be overcome by stimulation with αCD3 and αCD28 or immunodominant viral peptides.

Several reports have shown that IFNγ regulates the generation of IL-23-driven pathogenic Th17 cells producing IL-17 and GM-CSF. These Th17 cells have been implicated in several autoimmune disorders associated with dysregulated IM and neutrophil production [25, 26, 28]. Although a small population of IL-17^+^ γδ T cells were detected in the CLN (15%) and spleen (7%) at day 6 pi (Fig 4E and 4F: bottom row), we did not detect IL-17- or GM-CSF-secreting CD4 T cells in the BS, CLN or spleen (Fig 4E and 4F: top row, S4B–S4E and S6 Figs) of GKO mice. Moreover, depletion with either 3 or 8 doses of αGM-CSF mAb did not influence the survival of GKO mice, thereby excluding IL-17 and GM-CSF as candidate regulators of the neutrophilia observed in GKO mice (S7 Fig).

### Serum G-CSF and IL-6 are increased in the absence of IFNγ

**Fig 6:**
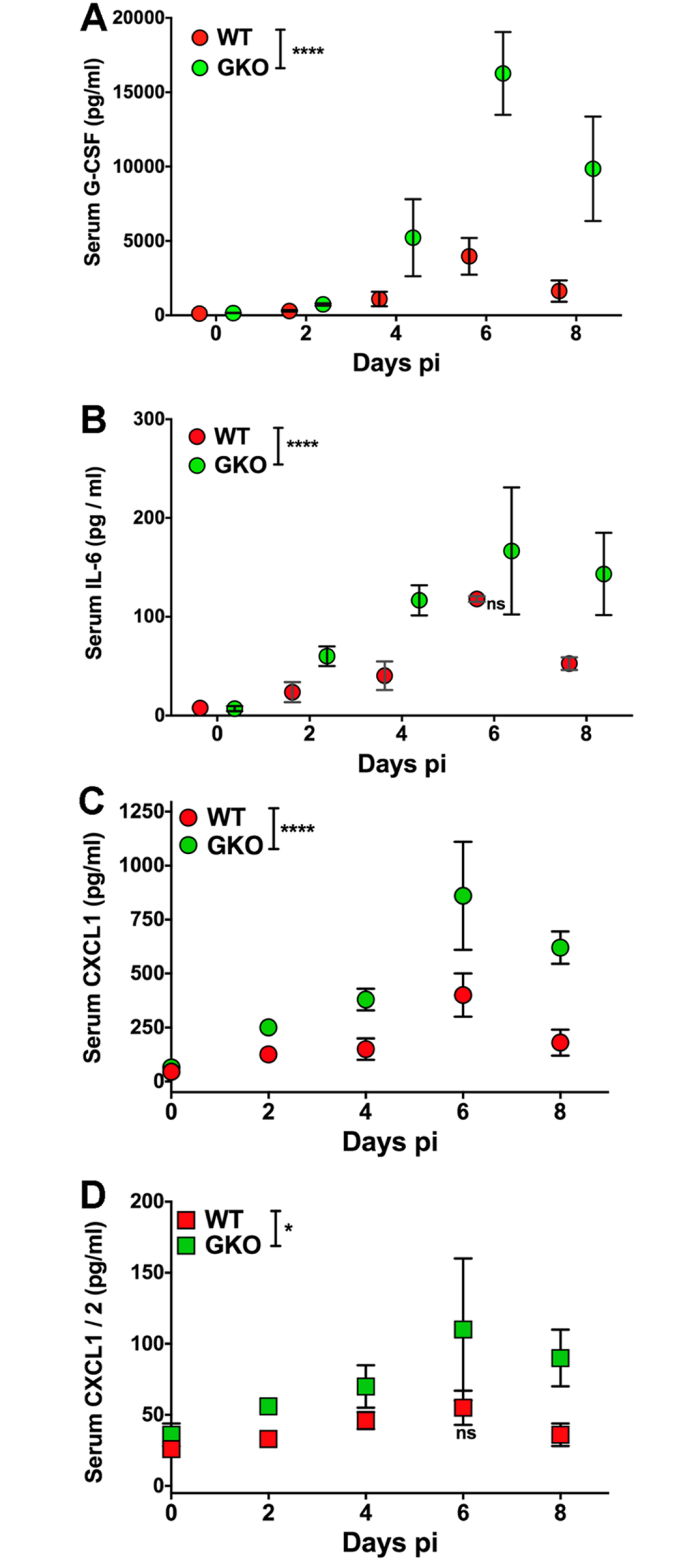
HSV infection of GKO mice elevates neutrophil related cytokines and chemokines. **(A)** G-CSF, **(B)** IL-6, **(C)** CXCL1 and **(D)** CXCL2 levels were determined in the sera of WT or GKO mice at the indicated time points using a multiplex ELISA (n = 3–6 mice per time point). G-CSF: ****p<0.0001 for days 4, 6 and 8 pi; IL-6- all time points except day 6 pi and CXCL1: ****p<0.0001; CXCL2- all time points except day 6 pi: *p = 0.02.

To determine the mechanism by which IFNγ controls neutrophil output from BM, we screened sera and BS obtained at various time points from infected GKO and WT mice for cytokines and chemokines (S1, S2 and S6 Figs). IL-17, IL-6, G-CSF and GM-CSF are potent inducers of neutrophils in the BM [6, 7, 26]. S6 Fig shows that many pro and anti-inflammatory cytokines and chemokines, including IFNα4, IL-4, IL-10, IL-17 and GM-CSF, were undetectable in the sera of infected GKO mice, consistent with the absence of Th17 and Th2 cells in the spleens and CLNs of these mice (Fig 4E and 4F and S4 Fig). IL-27, a potent inducer of IL-10 and Tr1 cells, was present at similar levels in the sera of both WT and GKO mice (S1 and S6 Figs) [48]. Nevertheless, IL-10 was undetectable in CD4 T cells isolated from BS, spleen or CLN of GKO mice, and both Tregs and Tr1 cells obtained from infected GKO mice were unable to protect GKO mice from HSV challenge (Fig 4D–4F and S4 Fig). The major cytokines upregulated in the sera and BS of infected GKO mice were IL-6 and G-CSF, along with the CXC chemokines CXCL1 (KC) and CXCL2 (MIP-2), which elicit neutrophil production and egress from BM **(**Fig 6A–6D, S2 and S6 Figs). Neutrophil and monocyte chemoattractants such as CCL3 (MIP-1α), CCL4 (MIP-1β) and CCL5 (RANTES) expression were also induced at high levels in BS but present at reduced levels in sera of GKO mice (S2 and S6 Figs). Astonishingly, G-CSF was increased ~3–4 fold in the sera of GKO mice compared to WT mouse sera (Fig 6A). Notably, although G-CSF levels declined in WT mice after day 6 pi (day 8 pi: < 1500 pg/ml), G-CSF levels remained extremely high in the sera of GKO mice (day 6 pi: ~15,000 pg/ml, and day 8 pi: ~10,000 pg/ml) (Fig 6A). Also, because the serum levels of G-CSF in GKO mice (735 pg/ml) were ~2-fold greater than in WT mice as early as day 2 pi (304 pg/ml) we speculate that control of G-CSF production was impaired at, or soon after, day 2 pi in GKO compared to WT mice.

### Depletion of G-CSF protects infected GKO mice from HSE

**Fig 7:**
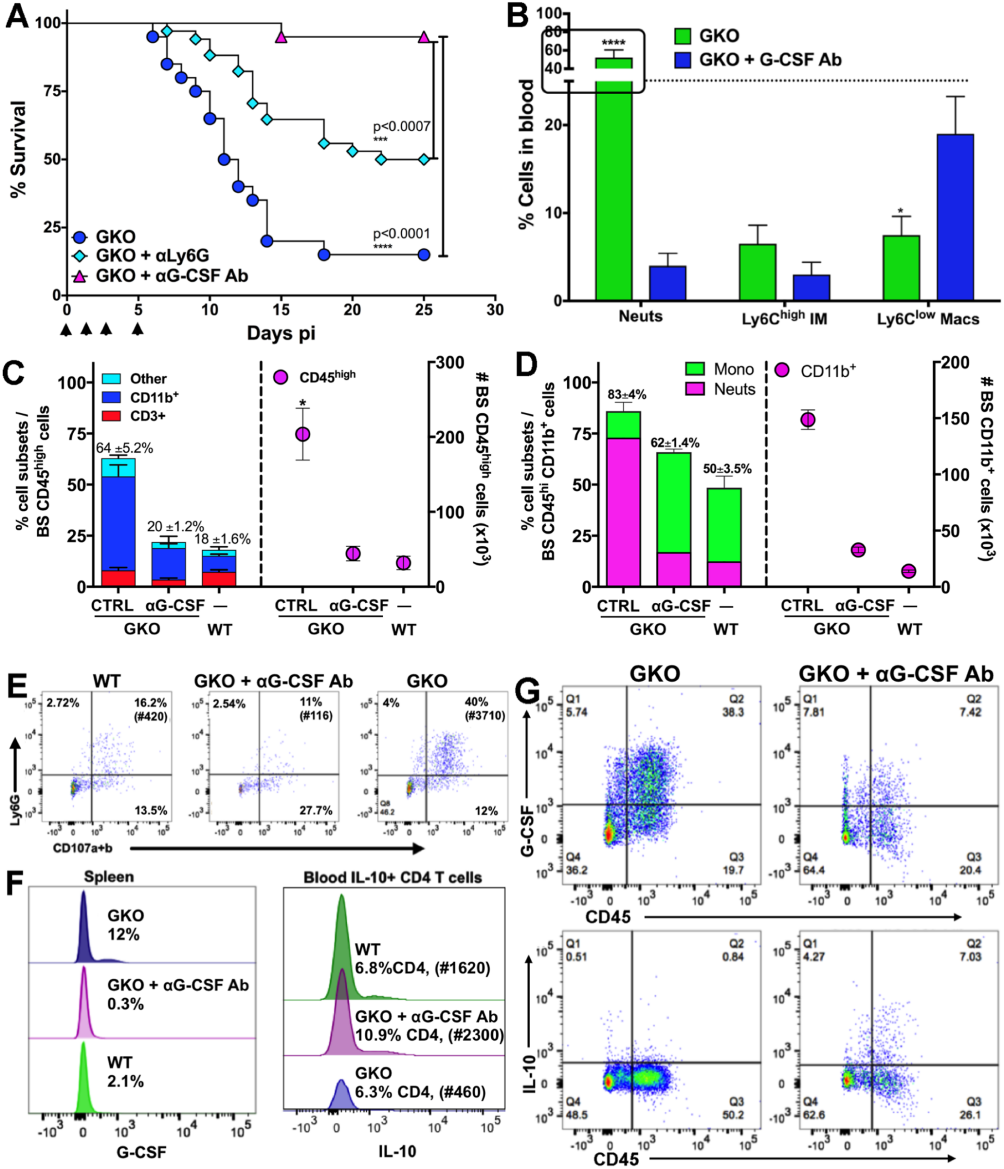
Depletion of G-CSF protects GKO mice by terminating neutrophil responses. **(A)** GKO mice were depleted of G-CSF or neutrophils with 3 doses of αG-CSF or αLy6G Ab (250 μg) respectively on days 0, 1 and 4 pi, and observed for survival following HSV infection. ***p<0.0007; ****p<0.0001. Data representative of 2 (for G-CSF)—3 (for Ly6G) experiments (n = 10–20 mice). (**B**) % Cell subsets isolated from blood mononuclear cells of GKO or αG-CSF-treated GKO mice at day 6 pi, ****p<0.0001, *p<0.05. (**C**) % (left y-axis) and # (right y-axis) of CD45^high^ cells in the BS of WT, GKO or αG-CSF Ab-treated GKO mice at day 6 pi. Numbers on top of bars indicate % CD45^high^ cells in BS and stacked bars within the CD45^high^ bar depict the proportions of CD3^+^ and CD11b^+^ or other cells. (**D**) % (left y-axis) and # (right y-axis) of CD11b^+^ cells within BS CD45^high^ infiltrates in WT, GKO and α G-CSF Ab-treated GKO mice. Numbers on top indicate % CD11b^+^ cells and stacked bars within the CD11b bar depict the proportions of SSC^low^ CD115^+^ monocytes / macrophages or SSC^high^ Ly6G^+^ neutrophils. **(E)** Percent degranulating (CD107a+b) Ly6G^+^ neutrophils within CD45^high^ cells isolated from the BS of WT, GKO and αG-CSF Ab-treated GKO mice at day 6 pi, # in parathesis indicate total number of degranulating PMN in the BS. (**F**) Intracellular staining for G-CSF by splenocytes (left plot) and IL-10 by CD4 T cells in the blood (right plot) of WT, GKO and αG-CSF Ab-treated GKO mice at day 6 pi. # in parenthesis indicate total number of IL-10 secreting CD4 T cells. **(G)** Intracellular G-CSF (top) and IL-10 (bottom) secretion by BS cells isolated from GKO (left) and αG-CSF Ab-treated GKO mice (right) at day 6 pi.

Depletion of Gr-1-expressing Ly6G^+^ neutrophils and Ly6C^high^ IMs in GKO mice, using an αGr1 mAb, did not protect the mice (S7 Fig). Also surprisingly, depletion of macrophages with chlodronate liposomes enhanced mortality in GKO mice, compared to PBS liposome-treated mice (S7 Fig). This result argues against macrophages or IM being causally involved in induction of HSE in GKO mice. Because neutrophils expanded more than other cell subsets in HSV-infected GKO mice, we depleted the neutrophils using a αLy6G-specific mAb. Remarkably, ~50% of the GKO mice survived HSV infection after Ly6G^+^ neutrophil depletion (Fig 7A), but the majority of mice died following cessation of mAb treatment. Because G-CSF was dramatically increased (~3–4 fold) in the sera of GKO mice, and G-CSFR is expressed predominantly by neutrophils and their progenitors in the BM, we depleted G-CSF using 3 doses of αG-CSF mAb to determine if reducing G-CSF levels could prevent neutrophilia and thus fatal encephalitis. As expected, G-CSF depletion protected the GKO mice from HSE (Fig 7A) with only one mouse succumbing on day 15 pi. Importantly, after G-CSF depletion, levels of circulating neutrophils in GKO mice were significantly reduced compared to untreated GKO mice, at days 4 (S8A and S8B Fig) and 6 pi (Fig 7B). CNS infiltration by CD45^high^ cells, Ly6C^high^ IMs, and Ly6G^+^ neutrophils was reduced in the G-CSF-depleted GKO mice, rendering them similar to WT mice (Fig 7C and 7D, S8C and S8D Fig). Similarly, G-CSF depletion drastically reduced the levels of degranulating neutrophils in the BS of GKO mice compared to the vastly increased numbers (~30X) in non-depleted GKO mice (Fig 7E). These data show that αG-CSF mAb treatment normalized the neutrophil and monocyte levels to that observed in WT mice, thus confirming that excessive levels of G-CSF drive neutrophillia in GKO mice. These data highlight the importance of G-CSF and IFNγ in balancing the neutrophil and monocyte output from the BM following infection with a viral pathogen.

### IL-10-mediated protection against HSE is restored by G-CSF inhibition

**Fig 8:**
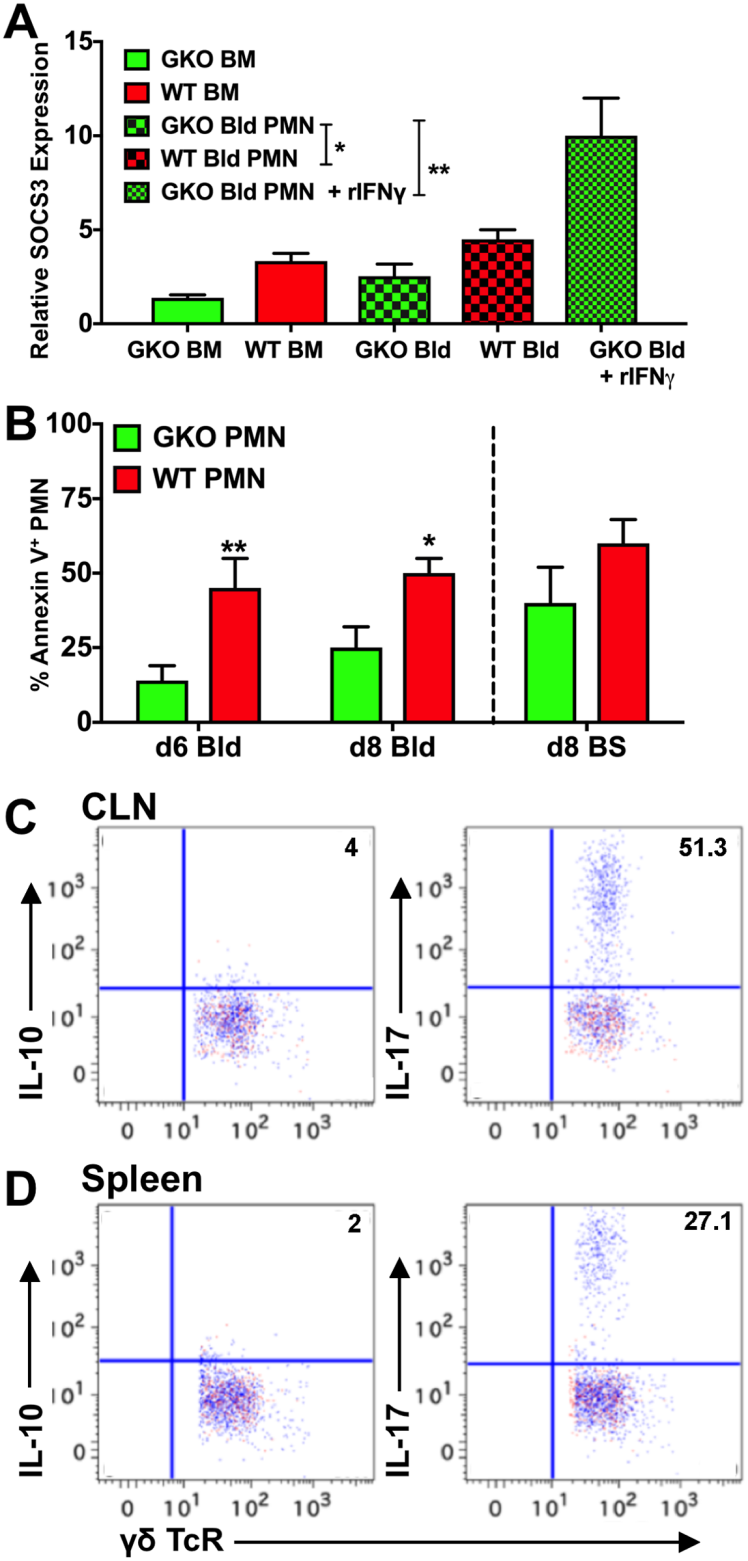
IFNγ controls neutrophil responses via apoptosis and SOCS3 expression. **(A)** Relative SOCS3 expression from Ly6G^+^ neutrophils (PMN) isolated from BM and blood (Bld) of WT and GKO mice at day 6 pi, as measured by RT-PCR; levels are relative to PMN isolated from uninfected mice. Blood-derived PMN treated with recombinant IFNγ in vitro for 30 min were compared to untreated neutrophils for SOCS3 expression. Data are representative of 2–3 experiments; *p = 0.013; **p = 0.0035. **(B)** Bar plots depict Annexin V^+^ PMN in the blood of WT and GKO mice at days 6 and 8 pi and BS at day 8 pi. Data are representative of 2 experiments with 2–3 mice per group. **p = 0.0011. (**C & D**) FACS plots show intracellular staining for IL-10 (left plots) and IL-17 (right plots) by γδ T cells isolated from **(C)** CLN and **(D)** spleen of GKO mice at day 14 pi. Red dots indicate no antigenic stimulation; blue dots indicate cells stimulated with PMA + ionomycin + HK-HSV.

Several cell types, including hematopoietic and non-hematopoietic cells, secrete G-CSF [11]. We found that CD11b^+^ cells, including macrophages and neutrophils in the blood and spleen (Fig 7F: left plot), secrete G-CSF in response to HSV infection. We found similar results for most cell types in the BS, including CD45^neg^ CD11b^-^ neural / glial cells, such as astrocytes, CD45^int^ CD11b^+^ microglia, and CD45^high^ CD11b^+^ monocytes and neutrophils (Fig 7G: top row). G-CSF secretion was greatly diminished by treatment of infected GKO mice with αG-CSF mAb (Fig 7F and 7G). Importantly, neutralizing G-CSF in GKO mice reinstated IL-10 secretion by ICOS^+^ CD4 T cells in the blood and BS (Fig 7F: right plot **and G**: bottom row) and reduced IM and neutrophil output from BM and infiltration into the BS (Fig 7D, S8A and S8B Fig). This result further validates the suppressive effects exerted by G-CSF induced GDSCs on T cell proliferation and cytokine secretion (Figs 5 and 7G). G-CSF is known to activate STAT3, thereby promoting neutrophil turnover and activation; however, SOCS3 mediated inhibition of STAT3 signaling curtails neutrophil expansion [18]. We observed that pSTAT3 expression was upregulated in neutrophils isolated from the blood of GKO mice at day 6 pi, compared to WT mice (S9A, S9B and S9C Fig). In contrast, SOCS3 expression was increased in neutrophils isolated from the blood and BM of WT mice, compared to GKO mice (Fig 8A). Futhermore, ex vivo treatment of neutrophils isolated at day 6 pi from blood of WT, GKO or G-CSF-depleted GKO mice with recombinant IFNγ, prior to treatment with recombinant G-CSF (S9B and S9C Fig), reduced pSTAT3 expression. Consistent with our in vivo results, GKO mouse blood-derived neutrophils, which were treated ex vivo with recombinant IFNγ, upregulated SOCS3 expression (Fig 8A). It was previously shown that in vitro treatment of naïve BM cells with recombinant IFN© upregulates SOCS3 expression, which in turn inhibits STAT3 signaling [49]. Our results extend this observation to an in vivo viral encephalitis model and show that IFN© is critical for regulation of G-CSF-mediated emergency hematopoiesis.

Since G-CSF is a survival factor for neutrophils, we asked whether GKO mice that have elevated G-CSF levels, exhibit reduced apoptosis of neutrophils. We found that indeed, the elevated G-CSF levels also increased the survival of circulating neutrophils as revealed by reduced Annexin V^+^ neutrophils in blood and BS of GKO, compared to αG-CSF treated GKO mice or WT mice, resulting in neutrophilia (Fig 8B and S8D Fig). These data are consistent with a previously described role for SOCS3 in suppression of G-CSF signaling and neutrophil apoptosis during emergency hematopoiesis [18]. Reduced neutrophil apoptosis and chronic inflammation have also been linked to increased IL-17 secretion. Although, we did not find CD4 T cells secreting IL-17 (Fig 4E and 4F), ~50% of the γδ T cells in the CLN and 30% in the spleen of GKO mice were IL-17^+^ at day 14 pi (Fig 8C and 8D) compared to 15% and 7% at day 6 pi in CLN and spleen respectively (Fig 4E and 4F). These data reveal a complex interaction network involving several pro- and anti-inflammatory cytokines that regulate CNS inflammation during viral infection. Our data support a model wherein a normal response to HSV infection depends on IFNγ-induced suppression of G-CSF and IL-6 levels (S10 Fig). This suppression of G-CSF allows for neutrophil apoptosis and the secretion of anti-inflammatory IL-10 from CD4 T cells, which controls IM expansion. In the absence of IFNγ, excessive neutrophil production and diminished IL-10 secretion predominate, leading to fatal HSE. This work demonstrates a mechanism by which IFNγ, a prototypical pro-inflammatory cytokine, can exert anti-inflammatory effects on innate inflammatory responses during viral infection of the CNS.

## Discussion

Dysregulated CNS inflammation is a hallmark of viral encephalitis. Our previous studies have shown an anti-inflammatory role for IL-10, which, by suppressing the generation of IMs, prevents HSE in WT mice [41, 42]. The present study uncovered a novel and complex interplay among IFNγ, G-CSF and IL-10, which determines the induction of neutrophila and HSE as shown schematically in S10 Fig. IFNγ was present at substantially greater levels than IL-10 in HSV-infected WT mice and played a unique role in the development and resolution of protective innate inflammatory responses in the CNS. Contrary to prior studies of neuroinflammatory diseases, we found that G-CSF and CXCL1, but not GM-CSF or IL-17, increased drammatically in HSV-infected GKO mice, resulting in invasion of the CNS by massive numbers of apoptosis-resistant, inflammatory neutrophils. Remarkably, we found that IFNγ controls G-CSF signaling by increasing SOCS3 expression in neutrophils and thereby induces apoptosis. Our study reveals that during viral encephalitis, it is G-CSF rather than GM-CSF that is the critical regulator of emergency hematopoiesis. The elevated levels of G-CSF induced production of GDSCs, which suppressed T-cell proliferation and function, including IL-10 secretion by regulatory T cells. Importantly, G-CSF depletion eliminated neutrophilia and restored protective IL-10 secreting regulatory T cells, thus preventing fatal HSE. GM-CSF has recently been ordained as a central mediator of tissue inflammation during emergency hematopoiesis and as a crucial conduit between tissue invading lymphocytes and myeloid cells [25, 30, 50]. However, our study reveals a novel anti-inflammatory role for IFNγ, where G-CSF but not GM-CSF suppression results in control of expansion and survival of potentially pathogenic neutrophils. Thus, the antagonistic G-CSF-IFNγ interactions emerge as a key regulatory node in control of the innate inflammatory response to virus infection of the CNS.

Neutrophils are the most abundant immune cell subset and are produced daily in prodigious amounts. Earlier studies established that neutrophils play indispensable roles in various aspects of host immunity, including defense against pathogens [2, 4, 12, 51]. Recent awareness that excessive numbers of neutrophils in tissues are associated with inflammatory diseases, has led to the re-evaluation of neutrophils as the primary drivers of inflammation associated pathology [2, 11, 13, 14, 52]. Consequently, neutrophil numbers and activity require precise regulation to avert the inflammatory pathology linked to various inflammatory diseases, including infections, autoimmunity and chronic disorders [11, 12, 52–55]. This regulation is imposed mainly by the G-CSFR / STAT3 / SOCS3 axis [18, 20, 56]. G-CSF is a key regulator of production, activation, and survival of neutrophils both during steady state and specifically during emergency hematopoiesis [56] and its levels are tightly controlled to avoid emergence of pathogenic neutrophils as a result of chronic G-CSF signaling [11]. We found that in contrast to WT mice, GKO mice were unable to suppress G-CSF levels after HSV infection, causing neutrophilia and death, demonstrating for the first time that IFNγ controls G-CSF levels and importantly neutrophil expansion and survival in vivo. In addition, several studies have documented the importance of GM-CSF and the Th17-related cytokines IL-23 and IL-17 for induction of inflammatory and autoimmune disease when IFNγ is lacking [25–28, 30]. In contrast, our data reveals that G-CSF, rather than GM-CSF, IL-23 or IL-17 is the primary cytokine that provokes neutrophilia and fatal HSE [52]. These results highlight the importance of IFNγ interaction with G-CSF signaling for regulation of neutrophil responses. We have identified a novel regulatory role for IFNγ as a suppressor of neutrophil proliferation during viral infection. Although IFNγ’s role in contraction of T-cell responses after the resolution of infections is well defined, its role in neutrophil contraction has been less clear. This work highlights the complexity of antiviral inflammatory responses, which depends on the virulence of the infectious agent and the extent of emergency hematopoiesis.

The resolution of an inflammatory response is initiated by apoptosis of neutrophils, which are then programed for phagocytosis by macrophages that release anti-inflammatory mediators to resolve inflammation [2]. However, during severe acute infections, apoptotic neutrophils can stimulate IL-17 secretion by either γδ or CD4 T cells, resulting in chronic inflammation [57]. Alternatively, reduced apoptosis of neutrophils, can sustain chronic inflammation [55]. Our results show that GKO mice have increased IL-17-secreting γδ T cells at day 14 pi, as well as reduced apoptosis of neutrophils, which likely accounts for impaired resolution of inflammation in GKO mice. Furthermore, depletion of G-CSF in GKO mice induced apoptosis of neutrophils and resolved inflammation, implicating impaired neutrophil apoptosis as the cause of chronic inflammation in the GKO mice.

Increased SOCS3 in neutrophils and BM cells is essential for desensitizing G-CSFR signaling to terminate neutrophil production [56]. Myeloid expression of SOCS3 is also crucial for controlling neuroinflammatory diseases [53, 58]. Our data show reduced SOCS3 expression in GKO, compared to WT, neutrophils, which likely impeded resolution of neutrophil responses, and is consistent with the established role for SOCS3 in regulating G-CSF signaling to prevent neutrophil-mediated disease.

We have previously shown that IL-10 suppressed expansion of IM in WT mice [59]. In our current study, excessive G-CSF in GKO mice was accompanied by deficient IL-10 production by CD4 T cells, which importantly was restored by neutralization of G-CSF. The mechanism linking IFNγ deficiency, G-CSF and lack of IL-10 production by CD4^+^ Tregs and Tr1 cells is intriguing and remains to be resolved. High levels of IL-27, which induce Tr1 cells, IL-10 production and suppression of Th17 cells [48, 60], were present in both WT and GKO mice, which explains the absence of Th17 cells but not the loss of IL-10 in GKO mice. In addition to the loss of IL-10 production, there are several possible reasons for the failure of GKO Tregs and Tr1 cells to protect from HSE, including effects of chronic inflammation in GKO mice, differences in TLR signaling or metabolic programs compared to WT Tregs and Tr1 cells [61, 62]. Also, the reduced SOCS3 expression, which resulted in sustained G-CSF signaling and neutrophil production, could contribute to IL-10 deficiency. Although IL-10 has been implicated in the resolution of neutrophil responses in a viral model of CNS infection [46], in our model IL-10KO mice displayed increased IM, but not neutrophil expansion; this difference likely results from the different virus-mouse strain combinations used in the two studies. Furthermore, IL-10KO T cells secreted increased IFNγ compared to WT T cells, which considered together with the reduced neutrophil levels in IL-10KO mice, clearly implicates IFNγ rather than IL-10 as an important regulator of neutrophils during inflammation. Thus our data reveals a “division of labor” for IL-10 and IFNγ in the resolution of inflammation following viral infection, with IL-10 being important for control of IMs and IFNγ being essential for termination of neutrophil responses.

IFNγ’s role in balancing the neutrophil-to-monocyte output from BM and regulation of neutrophil apoptosis during HSV infection, emphasizes its importance in controlling emergency hematopoiesis and neutrophil responses via regulation of G-CSF. Interestingly, a few studies have shown that the absence of type I IFNs results in CXCR2-driven neutrophil accumulation in the lungs or sensory ganglia following viral infections [63, 64]. Although we did not observe increased IFNα/β expression, increased expression of serum CXCL1 and CXCL2 which are CXCR2 ligands, was observed in GKO mice, compared to WT mice. Therefore, it is plausible that neutrophil expansion in the absence of IFNα/β [63, 64] might involve interference with the G-CSF / STAT3 / SOCS3 axis, similar to our results. Importantly, G-CSF signaling is crucial for the down-regulation of CXCR4, which permits neutrophil egress from BM [65]. The continued G-CSF signaling observed in GKO mice could account for the unabated production and egress of pathogenic neutrophils, and fatal HSE. Although the G-CSF receptor is present only on neutrophils, infiltration of other immune cells including IM into the BS of G-CSF depleted GKO mice was markedly reduced. Because neutrophils are the first cells to infiltrate an inflamed tissue, where by stimulating chemokine and integrin expression at the site of infection they enable other cells to enter the target organ, depletion of neutrophils likely resulted in reduced infiltration by macrophages along with all other cell types [66–68].

In various animal models of neutrophil-mediated inflammatory disease [13], administration of αG-CSF Ab resolves the disease by controlling neutrophil numbers and function. Thus, in the clinical setting, management of G-CSF levels with antibodies or analogous inhibitors could be beneficial in diseases where chronic inflammation is associated with aberrant inflammatory neutrophil responses. Our study corroborates a growing body of evidence emphasizing the importance of neutrophils in neuroinflammatory disorders, including viral-mediated encephalitis. We propose identifying novel factors that target the activation and function of neutrophils, rather than T cells, as a novel therapeutic approach for neuroinflammatory disease.

## Material and methods

### Mice, virus inoculation and antibody treatments

129S6 WT (129S6/SvEvTac) and Rag2^-/-^ (129S6/SvEvTac-Rag2tm1Fwa) mice were obtained from Taconic (Hudson, NY) while 129 IL-10KO (129(B6)-IL10tm1Cgn/J) mice were obtained from Jackson Laboratories (Bar harbor, Maine). 129 GKO mice were derived in this laboratory and have been described previously [45]. 129S6 WT, 129 GKO, 129 Rag2^-/-^ (abbreviated to Rag^-/-^) and 129 IL-10KO mice were bred in the Animal Research Facility at City of Hope. Both male and female mice, at 6–8 weeks of age, were infected with the HSV1 strain 17+ (3200 PFU) via corneal scarification, as previously described [41, 42]. To prevent increased mortality after infection, all mice received 4 mg intravenous immunoglobulin intraperitoneally at 24 h pi unless otherwise mentioned and were monitored daily for signs of encephalitis, as previously described [42]. Only WT recipients adoptively transferred with Tregs (Fig 4D) did not receive IVIG following HSV infection. G-CSF depletion was performed by administering 3 doses of 250 μg of αG-CSF Abs (R&D Systems) on days 0, 1 and 4 pi. 3 doses or 8 doses of αGM-CSF Ab (clone MP1-22E9) given every two days by ip injections was used to deplete GM-CSF, and its absence in serum confirmed by ELISA. Anti Gr-1 (clone RB6-8C5) was used to deplete Gr-1^+^ monocytes and neutrophils and anti-Ly6-G (clone 1A8) Abs to specifically deplete Ly6G^+^ neutrophils; depletion of neutrophils were determined by flow cytometry. Depletion of G-CSF using αG-CSF mAb was confirmed in serum by ELISA and its effects on neutrophil numbers were assessed by flow cytometry; ~ 90% of neutrophils were depleted in blood and ~85% in the BS. Virus titers in Tg and BS were determined by the plaque assay, as previously described [42].

### Isolation of mononuclear cells from the CNS and lymphoid organs

Isolation of mononuclear cells from spleen, CLN, blood and CNS have been described previously [42]. Briefly, brains, spinal cords, and BS were removed separately from mice perfused with PBS, minced, and digested with collagenase and DNase for 30 min prior to centrifugation at 800 x g for 25 min on a two-step Percoll gradient. Trigeminal ganglia were treated like CNS cells but instead of using a Percoll gradient, they were centrifuged at 400 x g for 10 min before passing through a nylon mesh. The purified cell fractions were then used for FACS. <5% CD45^high^ cells were present in the CNS of naïve and mock infected mice and these were mostly macrophages and DCs; very few T cells if any were detected. Day 6–8 pi time point was chosen to show cell infiltration into the BS as this time point represented peak infiltration by immune cells into the CNS of infected mice.

### Cell isolations and adoptive transfers

CD4^+^ or CD8^+^ T cells, CD25^+^ or ICOS^+^ CD4 T cells and CD11b^+^ monocytes and neutrophils, were isolated from spleen or blood of WT or GKO mice using cell-specific Ab tagged with magnet-conjugated nanobeads or by negative isolation EasySep kits according to the manufacturer’s instructions (Stemcell Technologies). Flow-sorted (FACSAria, BD Biosciences) CD11b^+^ Ly6G^+^ neutrophils isolated from blood were used for RT-PCR analysis. For adoptive transfers, CD25^+^ FoxP3 GFP^+^ Tregs (5x10^6^) or ICOS^+^ CD4 T cells (10^7^) were re-suspended in RPMI and injected intra-venously into the recipients prior to challenge with HSV 17+ by ocular scarification.

### Degranulation of neutrophils

Neutrophil and macrophage degranulation was determined in vitro in the absence of stimulation, or after stimulation of cells for 4–5 h with heat-killed HSV in the presence of αCD107a/b Abs to capture cell surface-associated LAMPs [42]. Resting macrophages did not express surface CD107a/b.

### Flow cytometric analysis

To determine cell surface expression, Ab-labeled cells were acquired on a BD Fortessa Analyzer (BD Biosciences, San Jose, CA) and flow cytometry analysis was performed using FlowJo software (Tree Star Inc.). Doublets were excluded from live cell populations. CD45 was used to distinguish BM-derived CD45^high^ leukocytes from CD45^int^ CD11b^+^ microglia and CD45^neg^ neural / glial cells as shown in Fig 3A. Mock infected mice contain < 5% CD45^high^ leukocytes in the BS. Neutrophils were determined by their SSC^high^, Ly6G^+^, CD115^-^ phenotype (Fig 3A and 3B). CD4^+^ Tregs were determined by reactivity to CD25 and intracellular FoxP3 expression (Fig 4A and 4B). Monocytes / macrophages were determined by a SSC^low^ CD115^+^ CD11b^+^ F480^+/low^ Ly6G^-^ phenotype, whereas IMs expressed high levels of Ly6C molecules (Fig 3A and 3B). Annexin reactivity to determine apoptosis in untreated *ex vivo* neutrophils was performed based on the manufacturer’s protocol using an Annexin V-specific Ab and 7-AAD (Biolegend Inc., San Diego, CA). Intracellular staining was performed as previously described [42]. Analysis of cytokine secretion during acute infection was done at day 6 pi because T cell responses were maximal between day 6–8 pi in the BS, CLN and spleen. Day 14 pi was chosen to study T cell responses following resolution of viral infection, because infectious virus could not be detected at this time point. Briefly, 10^6^ cells were stimulated for 4–6 h with or without peptide (CD8: gB_498-505_; CD4: heat killed (HK)-HSV; or PMA + ionomycin (Sigma-Aldrich) + HK-HSV, in the presence of protein transport inhibitors containing Brefeldin A or monensin (eBiosciences). Following FcR blocking, surface expression, using lineage specific antibodies, was determined. Then, the cells underwent fixation and membrane permeabilization using ebioscience IC fixation / permeabilization buffers (ebioscience), and the permeabilized cells were probed with α cytokine Abs to detect cytokines.

### Detection of phosphorylated STATs by FACS

pSTAT1 and pSTAT3 were determined using manufacturer’s protocol (BDPhosFlow, BD Biosciences, San Jose, CA) for mouse spenocytes. Briefly, mononuclear cells isolated from spleen, BM or blood were incubated with one of the following stimulations: recombinant IFNγ (20 ng/ml) for 2 h, recombinant G-CSF (5 ng/ml) for 20 min, PMA + ionomycin for 20 min or just medium control. In some cases, cells were treated first with recombinant IFNγ for 2 h, followed by recombinant G-CSF for 20 min. Following the stimulations, cells were fixed in pre-warmed 1x Lyse/fix buffer for 10–12 min at 37°C, permeabilized with chilled BD Perm Wash buffer III for 30 min, and probed for Ab specific for pSTAT1 and pSTAT3 and lineage specific surface markers (BD Biosciences, San Jose, CA).

### In vitro and ex vivo T cell suppression assay

CD11b^+^ Ly6G^+^ Neutrophils (PMN) and CD11b^+^ Ly6G^-^ Ly6C^+^ monocytes or APCs (Ly6G^-^ Ly6C^-^ CD11b^+^ / CD11c^+^ and B220^+^ cells) were isolated from blood of WT and GKO mice at day 6 pi because their levels were maximal at this time point; negative enrichment EasySep kits (Stemcell Tech., Vancouver BC) or flow sorting (FACSAria, BD Biosciences) was used for purification of cells. Memory T cells were obtained from spleens of HSV immunized WT mice at day 21 pi, while HSV specific effector T cells present in spleens of HSV infected WT and GKO mice at day 6 pi were used as effector cells. In some experiments (S5E and S5F Fig), memory CD4 and CD8 T cells were obtained from HSV infected WT and GKO mice at day 25 pi. CFSE labeled (5 μM) splenocytes or T cells (5x10^5^/well) and purified GKO or WT PMNs (suppressors; 5x10^4^) were cultured at 10:1 ratio in the presence or absence of HK-HSV (for CD4s and CD8s), H-2K^b^ HSV-1 gB_498-505_ peptide (for CD8s only) or with soluble α CD3 (2 μg/ml) and α CD28 (1 μg/ml) for 48–72 h to determine CD4 and CD8 T cell proliferation [47]. For ex vivo suppression of T cell proliferation, splenocytes isolated at day 6 or 8 pi were labeled with CFSE and cultured in the presence of HK-HSV for various times to determine suppression by neutrophils or IMs present within the respective groups (Fig 5). Since GKO spleens have increased numbers of neutrophils compared to WT spleens (9% Vs 5%), GKO PMN (Ly6G^+^ CD11b^+^) or monocyte / DCs (M/DC: CD11b^+^ Ly6G^-^/CD11c^+^) were added to the culture at a 1:10 (GKO innate cell: T cell) ratio along with CD19^+^ B cells in Fig 5D and 5E. Dilution of CFSE on labeled T cells revealed rates of proliferation at the different time points analyzed. Comparison with and without presence of suppressors in the presence or absence of antigen revealed the rate of suppression by neutrophils.

### Multiplex ELISA by luminex assay

Serum obtained from wt or GKO mice (n = 3 mice per group) at day 0 or different time pi were analyzed for a panel of 36 cytokines and chemokines using the ProCarta Plex Mouse Cytokine and Chemokine 36plex kit (Affymetrix eBioscience, San Diego, Ca), performed on the Bio-Rad Bio-Plex HTF System at the Clinical Immunobiology Correlative Studies Laboratory Core at City of Hope, Duarte, CA. We chose day 6 pi to show data for the majority of cytokines and chemokines in S1, S2 and S6 Figs as this time point reflected peak expression.

### RT^2^ profiler PCR array analysis of chemokine and cytokine gene expression

Total RNA was isolated from homogenized and lysed GKO and WT BS samples using the RNeasy Mini Kit (Qiagen, Valencia, CA) and following genomic DNA elimination, cDNA synthesized from mRNA using the RT^2^ first strand kit (Qiagen), according to manufacturer’s instructions. Chemokine and Cytokine gene expression was analyzed using Syber Green based RT^2^ Profiler PCR Arrays (PAMM-011z for Inflammatory Cytokines & Receptors) in a 96 well plate format. RT-PCR was performed using manufacturers protocol and analysis performed using the manufacturer provided Analysis template. Briefly, data analysis was performed based on the ΔΔC_T_ method, with normalization of raw data (Gene of interest = GOI) to 3–5 housekeeping genes (HKG) that were not been altered following infection (<1.5 difference in C_T_ value between day 0 and day 6 pi). The ΔΔC_T_ value was calculated by subtracting the averaged ΔC_T_ value of the GOI at day 6 pi BS (test group) from the ΔC_T_ value of the day 0 BS (control group) and the fold change (up- or down- regulation) calculated as 2^(-ΔΔC_T_).

### Statistics

Graph Pad Prism Software was used to analyze mortality data by the log rank (Mantel Cox or Gehan-Breslow-Wilcoxon) test, considering both the time of death and mortality rates. Statistical differences between groups of mice were calculated using two-way ANOVA with multiple pairwise comparisons (Turkeys or Sedaks) to determine the effects of time intervals between the different groups on cell populations and infiltrations or Student’s t tests for other calculations, with p ≤0.05 considered significant in the GraphPad Prism 6 software.

### Ethics statement

All animal procedures were performed in compliance with the City of Hope Beckman Research Institute Institutional Animal Care and Use Committee (IACUC) and within the framework of the Institute for Laboratory Animal Research (ILAR) Guide for the care and use of laboratory animals and all regulations of the United States Deparment of Agriculture (USDA) which implement the Animal Welfare Act (AWA) and the Public Health Service (PHS) Policy on Humane Care and Use of laboratory animals. All work done on mice used in this study was done under the approval of the IACUC of the City of Hope Beckman Research Institute that reviewed and approved the relevant protocol #07043.

## Supporting information

S1 Fig IFNγ induced chemokines are induced in WT mice. Serum obtained from WT mice at day 6 pi were analyzed for cytokines and chemokines by a multiplex ELISA based luminex assay. Data is represented as relative to day 0 levels for the cytokine or chemokine (n = 3 mice).
(TIF)

S2 Fig Neutrophil and monocyte related chemokines are upregulated in BS of GKO mice. (**A**) Chemokine and (**B**) Cytokine expression was analyzed in BS of WT and GKO mice at day 6 pi by RT^2^ Profiler PCR arrays (for Inflammatory Cytokines, Chemokines & Receptors) (n = 3 mice / group). Gene expression of cytokines and chemokines in the BS at day 0 or day 6 pi were normalized to housekeeping genes and the data is represented as fold change (up or down-regulation) at day 6 pi relative to day 0 BS using the ΔΔCT method (see Methods).
(TIF)

S3 Fig Virus titers and opposing effects of IFNγ and IL-10 on monocytes and neutrophils. **(A)** Virus titers in the trigeminal ganglia (Tg) of WT and GKO mice at indicated time points as determined by plaque assay (n = 5–6 mice per time point). **(B)** CD45^high^ infiltrating cells in the brain (Brn) and spinal cords (SC) of WT and GKO mice. Data is representative of 2 (for GKO)– 3 (for WT) experiments (n = 4–6 mice). **(C)** Ratio of neutrophils to IM in the blood of IL-10KO and Rag^-/-^ mice at indicated time points. **(D)** BS CD45^high^ CD4 T cells isolated from HSV infected IL-10KO mice on day 6 pi were probed for IFNγ (left plot) and IL-17 (right plot) by intracellular flow cytometry following antigeni stimulation. Representative FACS plots show cells gated on BS CD45^high^ cells.
(TIF)

S4 Fig Functional status of CD4 and CD8 T cells in GKO mice. (**A**) Spleen cells isolated at day 6 pi from GKO mice were probed for various Treg markers. Representative flow cytometry plots gated on splenic CD4 T cells depicting expression of FoxP3 in blue or isotype in red (left plot), CD25 and FoxP3 (middle plot) and ICOS and FoxP3 (right plot). **(B)** Representative flow cytometry plots gated on splenic CD4 T cells isolated from GKO mice at day 6 pi showing intracellular IL-10 and IL-17 (left plot) or TNF-α and IL-4 (right plot). Antigen stimulated cells shown as blue dots and un-stimulated cells as red dots. **(C-D)** BS mononuclear cells isolated from GKO mice on **(C)** day 6 or **(D)** day 14 pi probed by ICS following antigen stimulation for IL-17 and IL-10 expression: IL-17 expression by CD3^+^ T cells (left plot gated on CD45^high^ cells) and CD4 T cells (middle plot gated on CD45^high^ CD3^+^ T cells); IL-10 expression by CD11b^+^ cells (right plot gated on CD45^high^ cells). **(E)** GM-CSF (left plot) following antigen stimulation or FoxP3 (right plot) expressing CD4 T cells in the BS of GKO mice at day 14 pi (plots gated on CD45^high^ CD3^+^ T cells).
(TIF)

S5 Fig GKO GDSCs suppress effector and memory T cell proliferation. Spleen cells isolated from HSV infected WT or GKO mice at day 6 pi (as in Fig 5) were labeled with CFSE and stimulated with HK-HSV to determine effector (e) CD4 and CD8 T cell proliferation. Shown in **A** are representative FACS plots at 4 h post culture for undivided WT eCD4 (left plot: gated on WT CD4 T cells) and WT H-2K^b^ HSV-1 gB_498-505_ tetramer^+^ eCD8 (right plot: gated on WT CD8) T cells. **(B-C)**, Ly6G^+^ neutrophils (PMN) isolated from the blood of HSV infected WT (left plot) or GKO (right plot) mice at day 6 pi were cultured with CFSE labeled memory (m) **(B)** CD4 or **(C)** CD8 T cells obtained from spleens of HSV immunized WT mice in the presence or absence of heat killed HSV (HK-HSV: for CD4 and CD8) or **(D)** H-2K^b^ HSV-1 gB_498-505_ peptide (for CD8 T cells only) to determine suppression of T cell proliferation; after culturing for 72 h, T cells were analyzed by flow cytometry for dilution of CFSE (indicative of proliferation) and presence of high surface expression of PD-1 molecules (% denoted above box) indicating an inhibitory phenotype. Intermediate PD-1 expression representing activated T cells is not included in the boxed area. Percentages in parenthesis denote cells having undergone more than one division. **(E)** Memory (m) CD4 and **(F)** CD8 T cells isolated (at day 25 pi) from spleens of HSV infected WT (left plot) or GKO (right plot) mice cultured in the presence of Ly6G^+^ neutrophils (PMN) obtained from blood of HSV infected GKO mice at day 6 pi as described above in **B-C** were stimulated in the presence (red) or absence (blue) of αCD3 and αCD28 for 48 h prior to analysis of CFSE dilution indicative of proliferation; top bar indicates > 1 division and bottom bar indicates cells > 2 divisions. All stimulation conditions are labeled in blue.
(TIF)

S6 Fig Absence of IFNγ absence induces neutrophil related chemokines and cytokines. Serum obtained from HSV GKO mice at day 6 pi were analyzed for cytokines and chemokines by a multiplex ELISA based luminex assay. Data is represented as relative to day 0 levels (obtained from uninfected GKO mice) for the cytokine or chemokine (n = 3 mice).
(TIF)

S7 Fig GM-CSF or Gr-1 depletion does not protect from neutrophilia. HSV infected GKO mice were treated from day 0 pi with three doses of the indicated Abs (250 μg) on days 0, 1 and 4 pi and monitored for survival (n = 8–12 per group). Some mice received 8 doses of αGM-CSF Ab spaced two days apart while others received only three doses on days 0, 1 and 4 pi. Two groups of mice received one dose of chlodronate or PBS liposome at day 0 pi.
(TIF)

S8 Fig G-CSF ablation inhibits neutrophil invasion of the CNS. **(A)** Cells isolated at day 4 pi from blood of HSV infected WT, GKO or αG-CSF Ab treated GKO mice were analyzed for SSC^high^ CD11b^+^ neutrophils or **(B)** CD115^+^ Ly6C^high^ monocytes and CD115^-^ Ly6C^int^ PMN. (**C**) Mononuclear cells isolated from BS of HSV infected HSV infected WT, G-CSF depleted GKO or untreated GKO mice at day 4 pi were analyzed for CD45 and CD11b expression; **(D)** CD45^high^ gated BS cells analyzed for CD11b^+^ SSC^high^ neutrophils and SSC^low^ monocytes.
(TIF)

S9 Fig G-CSF sustains STAT3 signaling in the absence of IFNγ. **(A)** Mononuclear cells isolated from blood of HSV infected WT, GKO or αG-CSF Ab treated GKO mice at day 6 pi were probed for surface expression Ly6G/Gr-1 and CD11b, **(B)**, phospho STAT3 (pSTAT3) expression in Gr-1/Ly6G^high^ CD11b^+^ CD115^-^ SSC^high^ neutrophils isolated from blood of HSV infected GKO mice at day 6 pi as detected by phosphoflow; after a 2 h *in vitro* treatment with (red) or without (blue) recombinant IFNγ followed by recombinant G-CSF for 20 min, or without any treatment (CTRL: gray) and **(C)**, Ly6G^+^ neutrophils isolated from the blood of the three groups of HSV infected mice at day 6 pi were analyzed for increase in mean fluorescence intensity (MFI) of pSTAT3 expression following *in vitro* treatments with (1) recombinant IFNγ for 2 h followed by recombinant G-CSF for 20 min or (2) recombinant G-CSF alone, relative to no treatment. **(D)** Annexin V reactive Ly6G^+^ neutrophils in the blood of HSV infected wt, GKO or α G-CSF treated GKO mice at day 6 pi.
(TIF)

S10 Fig Excessive G-CSF causes neutrophilia. Top panel: GKO mice. In the absence of IFNγ, copious amounts of G-CSF and CXCL1 are secreted by cells in the brain including microglia, astrocytes and macrophages. Excessive G-CSF provokes neutrophilia in the bone marrow and these apoptosis-resistant neutrophils along with Ly6C^high^ inflammatory monocytes (IM) invade the brainstem in massive numbers inflicting damage that culminates in fatal HSV encephalitis (HSE). G-CSF also suppresses IL-10 production by regulatory CD4 T cells resulting in an inability to regulate IM. Bottom panel: WT mice. IFNγ produced by WT T cells suppresses G-CSF production via increased SOCS3 expression thereby inducing neutrophil apoptosis and protecting WT mice from fatal HSE. Cells involved in this mechanism are shown in the key. Red arrows: inhibitory, green arrows: stimulatory; text in gray and dashed arrows indicate reduced or absent effectors.
(TIF)
