# Supplementary figures and images for: IFNγ inhibits G-CSF induced neutrophil expansion and invasion of the CNS to prevent viral encephalitis
Source: PLoS Pathog. 2018 Jan 19;14(1):e1006822. doi: 10.1371/journal.ppat.1006822 (PMC5792029; doi:10.1371/journal.ppat.1006822)

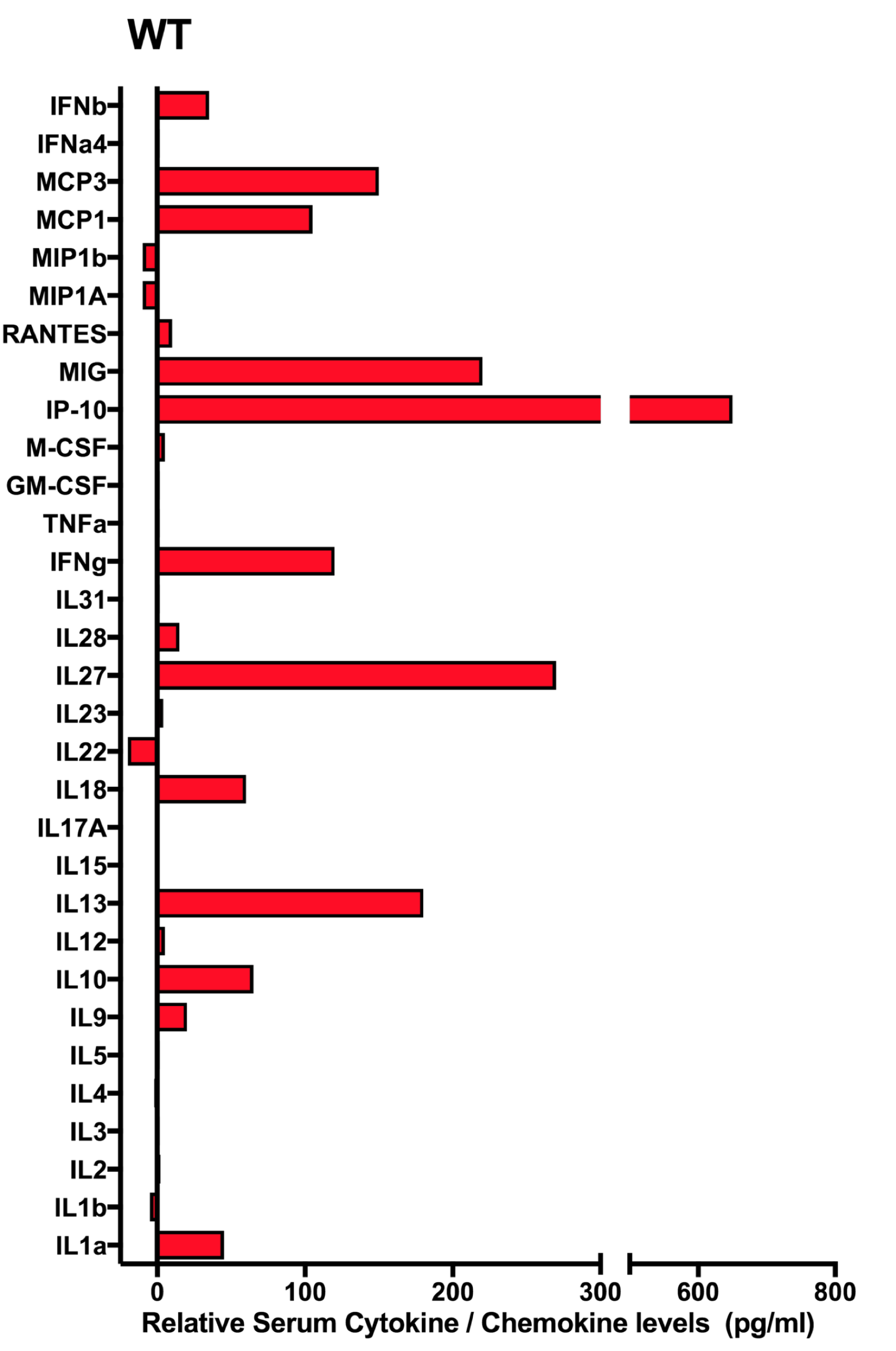

Supplement: S1 Fig — Serum obtained from WT mice at day 6 pi were analyzed for cytokines and chemokines by a multiplex ELISA based luminex assay. Data is represented as relative to day 0 levels for the cytokine or chemokine (n = 3 mice). (TIF) [file ppat.1006822.s001.tif]

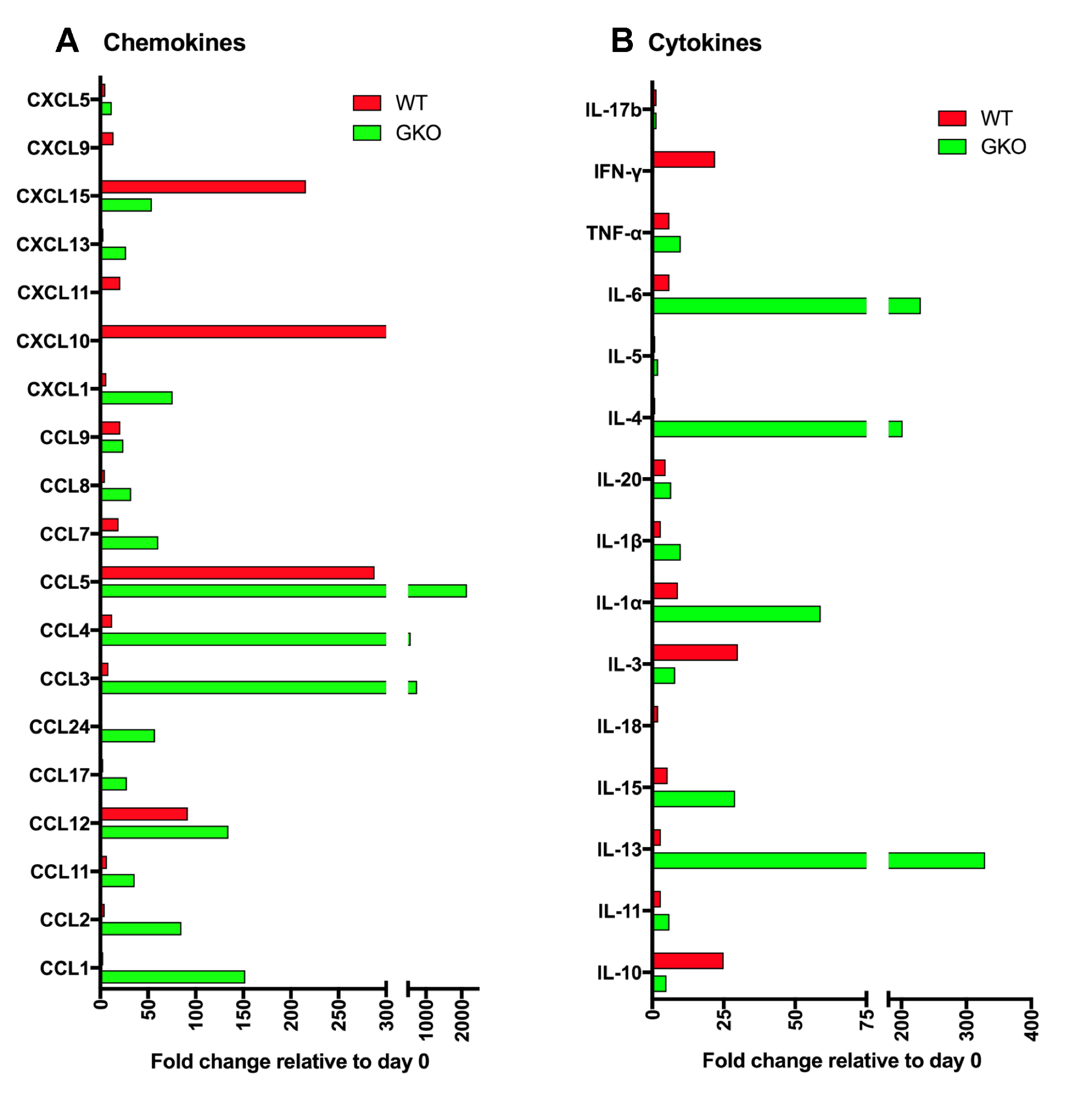

Supplement: S2 Fig — (A) Chemokine and (B) Cytokine expression was analyzed in BS of WT and GKO mice at day 6 pi by RT2 Profiler PCR arrays (for Inflammatory Cytokines, Chemokines & Receptors) (n = 3 mice / group). Gene expression of cytokines and chemokines in the BS at day 0 or day 6 pi were normalized to housekeeping genes and the data is represented as fold change (up or down-regulation) at day 6 pi relative to day 0 BS using the ΔΔCT method (see Methods). (TIF) [file ppat.1006822.s002.tif]

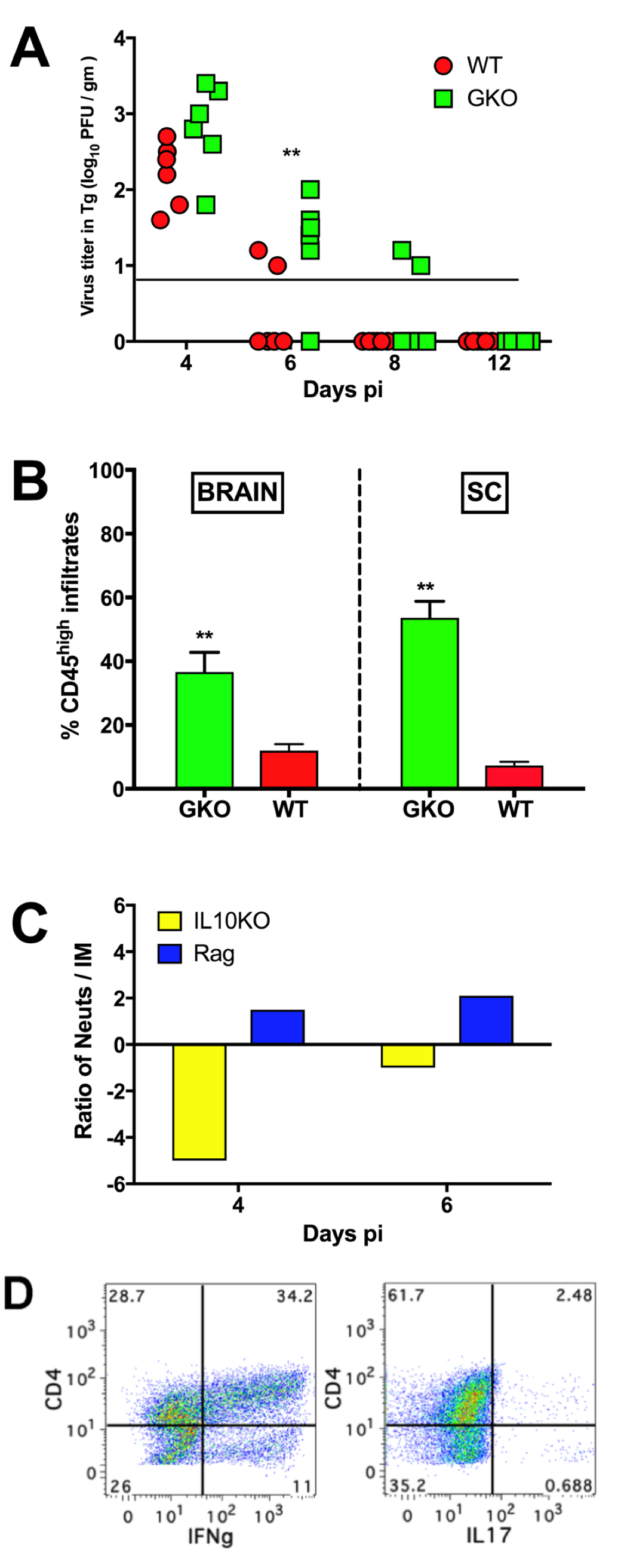

Supplement: S3 Fig — (A) Virus titers in the trigeminal ganglia (Tg) of WT and GKO mice at indicated time points as determined by plaque assay (n = 5–6 mice per time point). (B) CD45high infiltrating cells in the brain (Brn) and spinal cords (SC) of WT and GKO mice. Data is representative of 2 (for GKO)– 3 (for WT) experiments (n = 4–6 mice). (C) Ratio of neutrophils to IM in the blood of IL-10KO and Rag-/- mice at indicated time points. (D) BS CD45high CD4 T cells isolated from HSV infected IL-10KO mice on day 6 pi were probed for IFNγ (left plot) and IL-17 (right plot) by intracellular flow cytometry following antigeni stimulation. Representative FACS plots show cells gated on BS CD45high cells. (TIF) [file ppat.1006822.s003.tif]

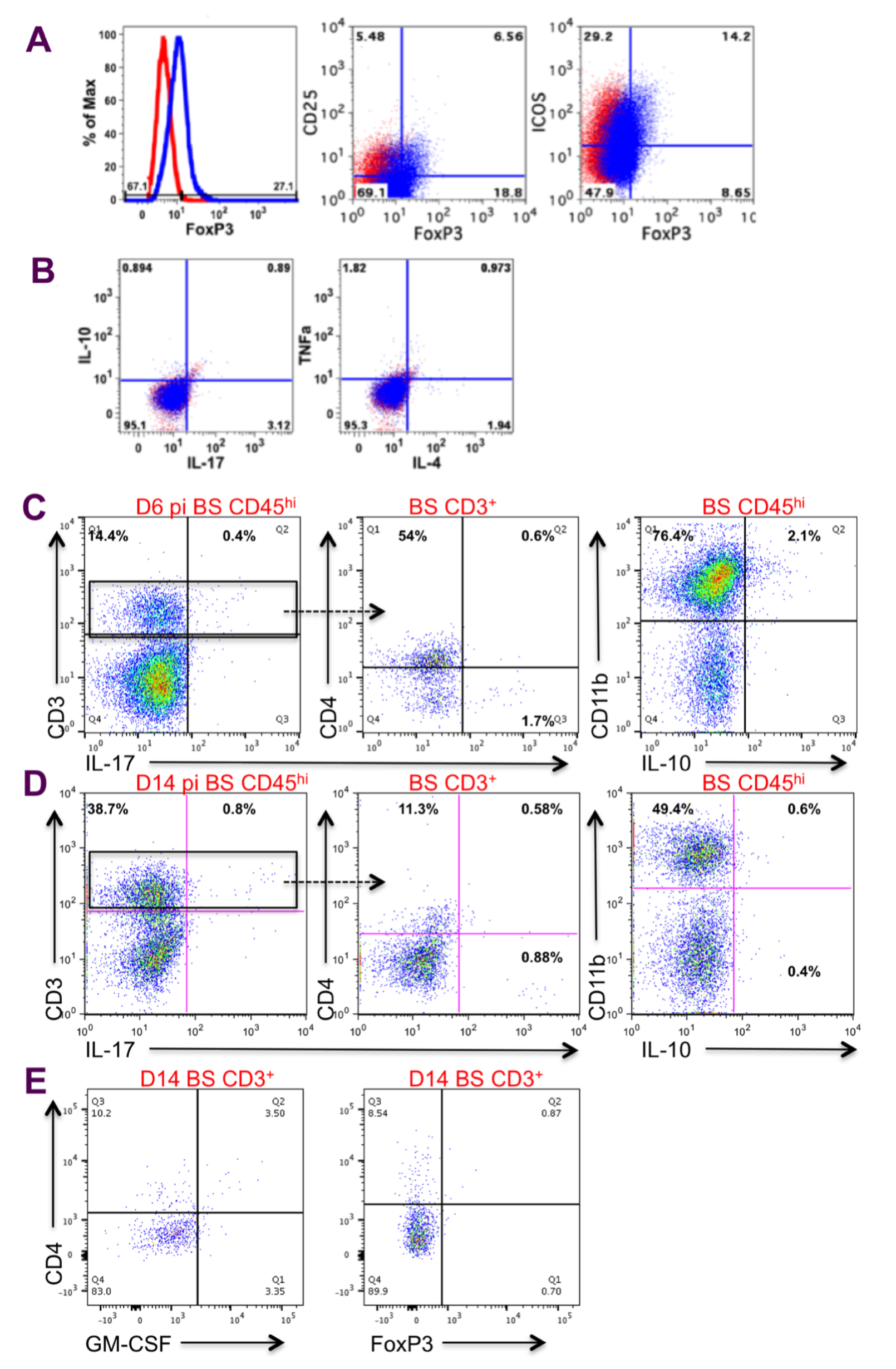

Supplement: S4 Fig — (A) Spleen cells isolated at day 6 pi from GKO mice were probed for various Treg markers. Representative flow cytometry plots gated on splenic CD4 T cells depicting expression of FoxP3 in blue or isotype in red (left plot), CD25 and FoxP3 (middle plot) and ICOS and FoxP3 (right plot). (B) Representative flow cytometry plots gated on splenic CD4 T cells isolated from GKO mice at day 6 pi showing intracellular IL-10 and IL-17 (left plot) or TNF-α and IL-4 (right plot). Antigen stimulated cells shown as blue dots and un-stimulated cells as red dots. (C-D) BS mononuclear cells isolated from GKO mice on (C) day 6 or (D) day 14 pi probed by ICS following antigen stimulation for IL-17 and IL-10 expression: IL-17 expression by CD3+ T cells (left plot gated on CD45high cells) and CD4 T cells (middle plot gated on CD45high CD3+ T cells); IL-10 expression by CD11b+ cells (right plot gated on CD45high cells). (E) GM-CSF (left plot) following antigen stimulation or FoxP3 (right plot) expressing CD4 T cells in the BS of GKO mice at day 14 pi (plots gated on CD45high CD3+ T cells). (TIF) [file ppat.1006822.s004.tif]

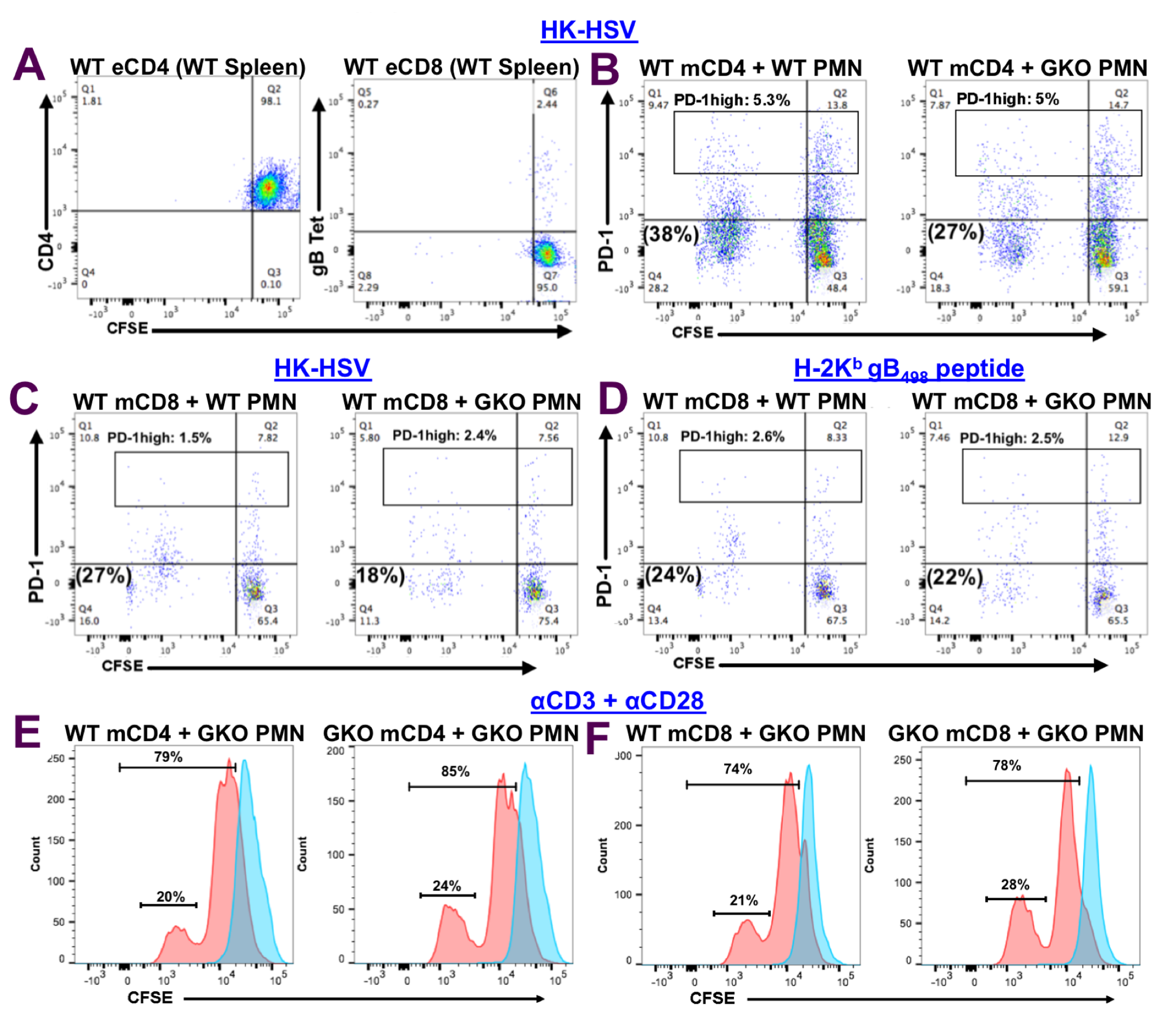

Supplement: S5 Fig — Spleen cells isolated from HSV infected WT or GKO mice at day 6 pi (as in Fig 5) were labeled with CFSE and stimulated with HK-HSV to determine effector (e) CD4 and CD8 T cell proliferation. Shown in A are representative FACS plots at 4 h post culture for undivided WT eCD4 (left plot: gated on WT CD4 T cells) and WT H-2Kb HSV-1 gB498-505 tetramer+ eCD8 (right plot: gated on WT CD8) T cells. (B-C), Ly6G+ neutrophils (PMN) isolated from the blood of HSV infected WT (left plot) or GKO (right plot) mice at day 6 pi were cultured with CFSE labeled memory (m) (B) CD4 or (C) CD8 T cells obtained from spleens of HSV immunized WT mice in the presence or absence of heat killed HSV (HK-HSV: for CD4 and CD8) or (D) H-2Kb HSV-1 gB498-505 peptide (for CD8 T cells only) to determine suppression of T cell proliferation; after culturing for 72 h, T cells were analyzed by flow cytometry for dilution of CFSE (indicative of proliferation) and presence of high surface expression of PD-1 molecules (% denoted above box) indicating an inhibitory phenotype. Intermediate PD-1 expression representing activated T cells is not included in the boxed area. Percentages in parenthesis denote cells having undergone more than one division. (E) Memory (m) CD4 and (F) CD8 T cells isolated (at day 25 pi) from spleens of HSV infected WT (left plot) or GKO (right plot) mice cultured in the presence of Ly6G+ neutrophils (PMN) obtained from blood of HSV infected GKO mice at day 6 pi as described above in B-C were stimulated in the presence (red) or absence (blue) of αCD3 and αCD28 for 48 h prior to analysis of CFSE dilution indicative of proliferation; top bar indicates > 1 division and bottom bar indicates cells > 2 divisions. All stimulation conditions are labeled in blue. (TIF) [file ppat.1006822.s005.tif]

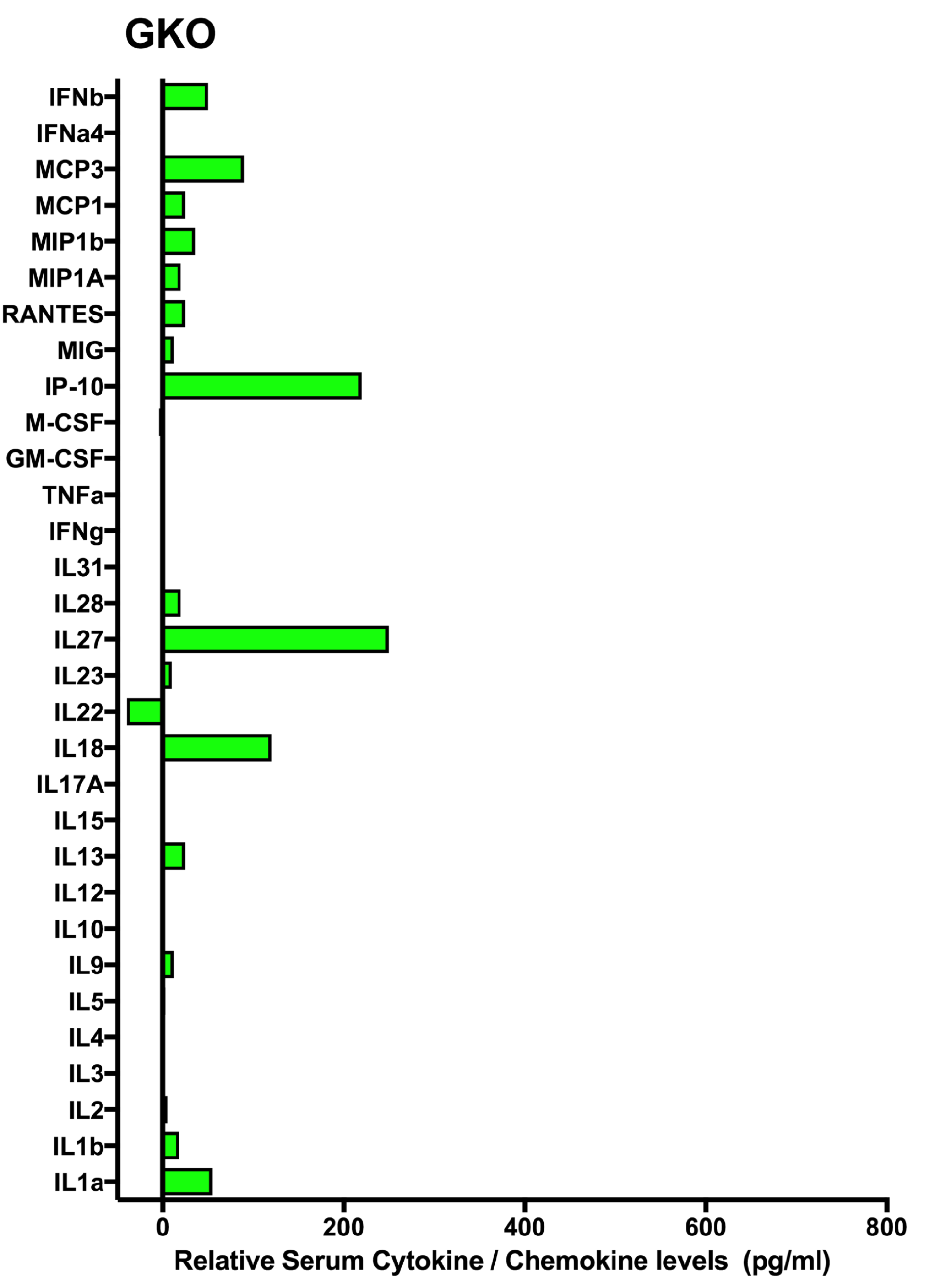

Supplement: S6 Fig — Serum obtained from HSV GKO mice at day 6 pi were analyzed for cytokines and chemokines by a multiplex ELISA based luminex assay. Data is represented as relative to day 0 levels (obtained from uninfected GKO mice) for the cytokine or chemokine (n = 3 mice). (TIF) [file ppat.1006822.s006.tif]

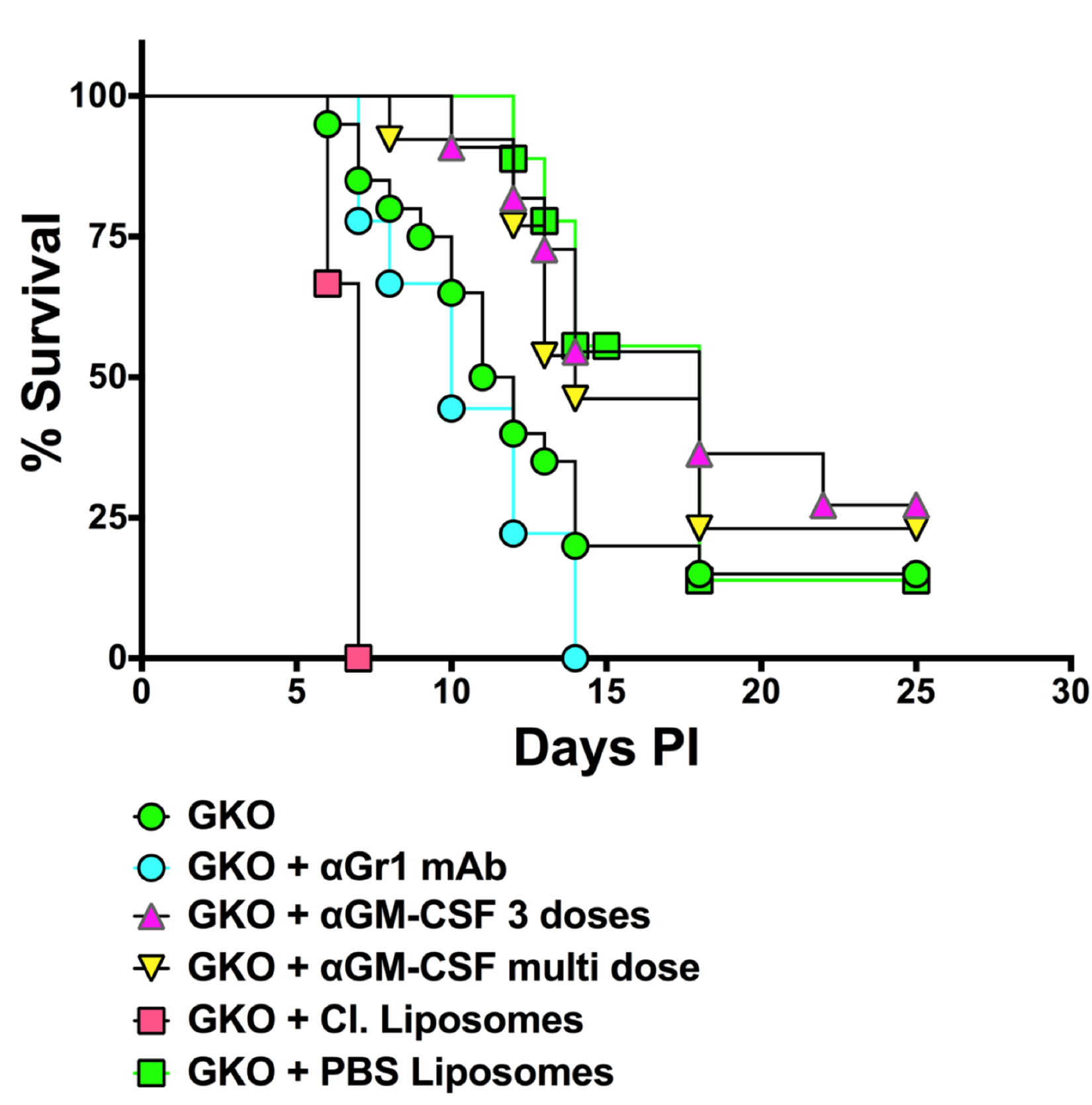

Supplement: S7 Fig — HSV infected GKO mice were treated from day 0 pi with three doses of the indicated Abs (250 μg) on days 0, 1 and 4 pi and monitored for survival (n = 8–12 per group). Some mice received 8 doses of αGM-CSF Ab spaced two days apart while others received only three doses on days 0, 1 and 4 pi. Two groups of mice received one dose of chlodronate or PBS liposome at day 0 pi. (TIF) [file ppat.1006822.s007.tif]

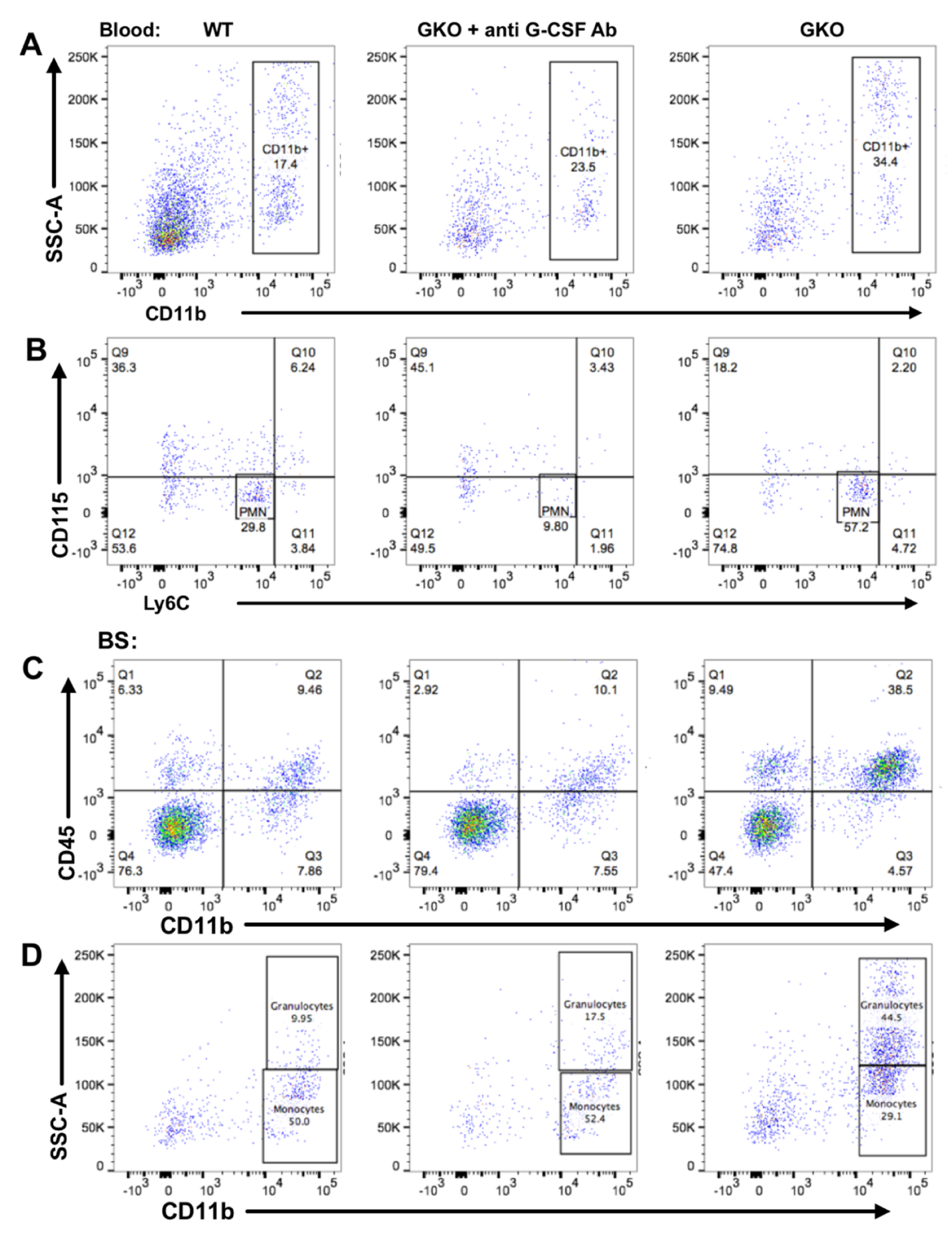

Supplement: S8 Fig — (A) Cells isolated at day 4 pi from blood of HSV infected WT, GKO or αG-CSF Ab treated GKO mice were analyzed for SSChigh CD11b+ neutrophils or (B) CD115+ Ly6Chigh monocytes and CD115- Ly6Cint PMN. (C) Mononuclear cells isolated from BS of HSV infected HSV infected WT, G-CSF depleted GKO or untreated GKO mice at day 4 pi were analyzed for CD45 and CD11b expression; (D) CD45high gated BS cells analyzed for CD11b+ SSChigh neutrophils and SSClow monocytes. (TIF) [file ppat.1006822.s008.tif]

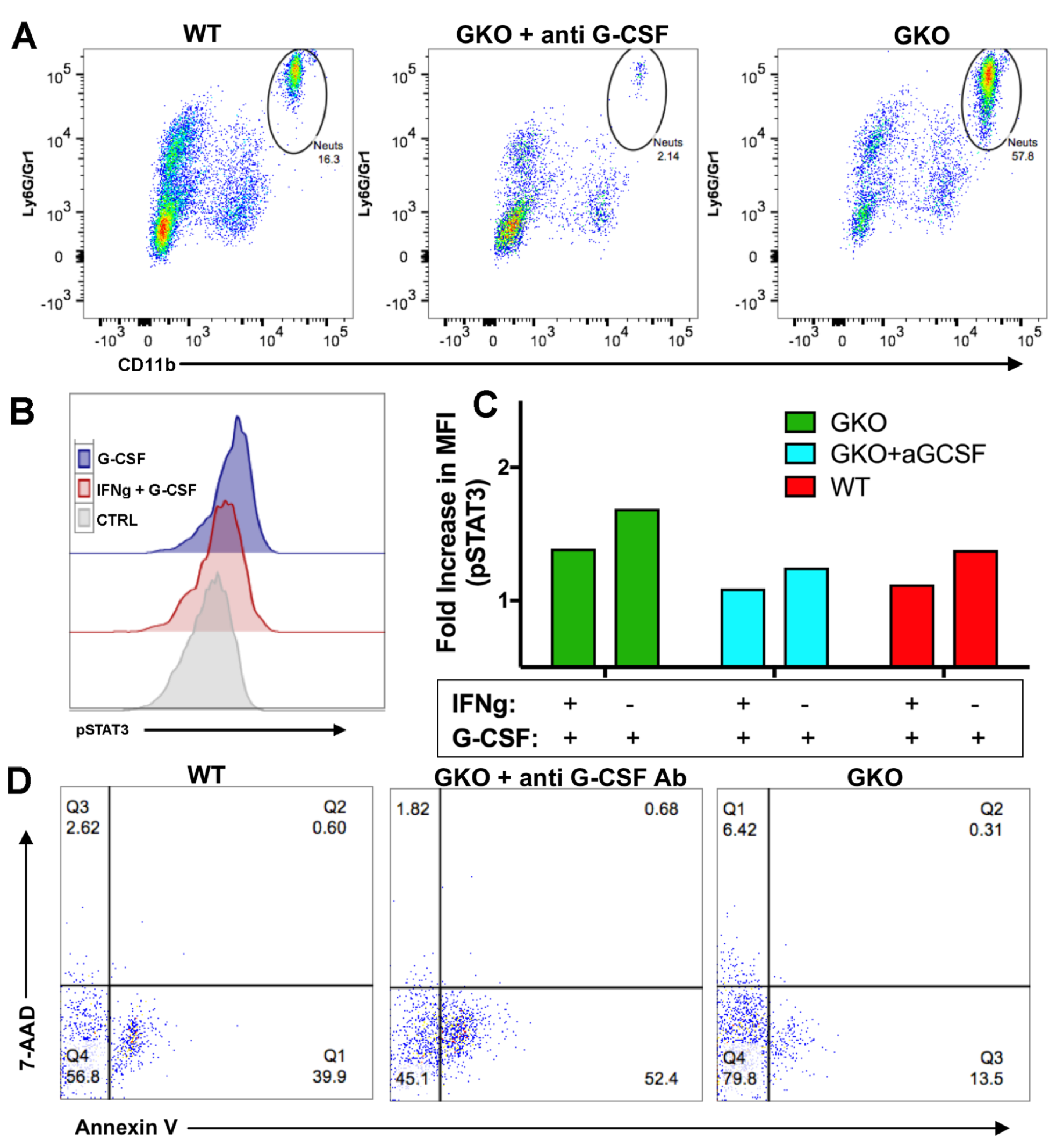

Supplement: S9 Fig — (A) Mononuclear cells isolated from blood of HSV infected WT, GKO or αG-CSF Ab treated GKO mice at day 6 pi were probed for surface expression Ly6G/Gr-1 and CD11b, (B), phospho STAT3 (pSTAT3) expression in Gr-1/Ly6Ghigh CD11b+ CD115- SSChigh neutrophils isolated from blood of HSV infected GKO mice at day 6 pi as detected by phosphoflow; after a 2 h in vitro treatment with (red) or without (blue) recombinant IFNγ followed by recombinant G-CSF for 20 min, or without any treatment (CTRL: gray) and (C), Ly6G+ neutrophils isolated from the blood of the three groups of HSV infected mice at day 6 pi were analyzed for increase in mean fluorescence intensity (MFI) of pSTAT3 expression following in vitro treatments with (1) recombinant IFNγ for 2 h followed by recombinant G-CSF for 20 min or (2) recombinant G-CSF alone, relative to no treatment. (D) Annexin V reactive Ly6G+ neutrophils in the blood of HSV infected wt, GKO or α G-CSF treated GKO mice at day 6 pi. (TIF) [file ppat.1006822.s009.tif]

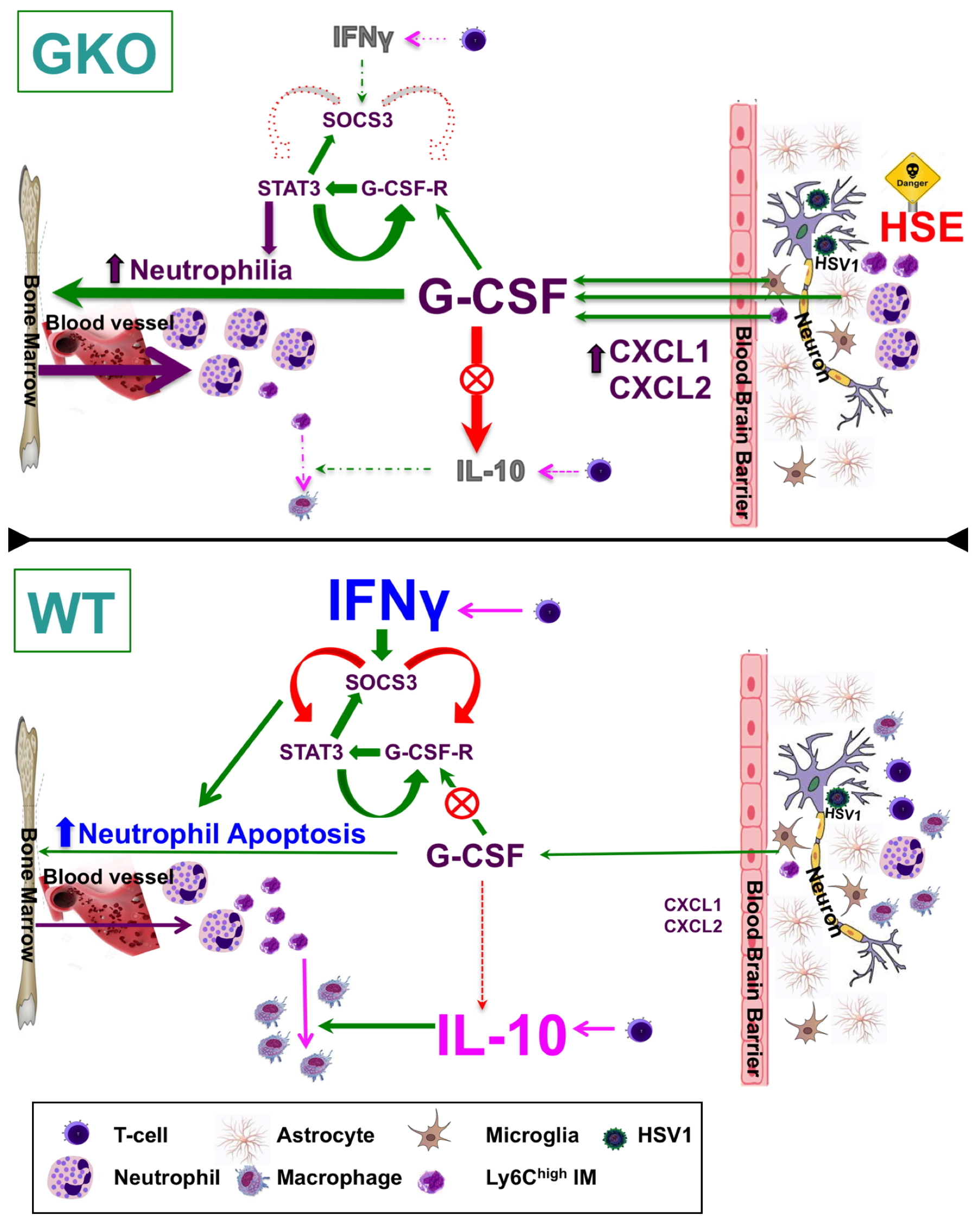

Supplement: S10 Fig — Top panel: GKO mice. In the absence of IFNγ, copious amounts of G-CSF and CXCL1 are secreted by cells in the brain including microglia, astrocytes and macrophages. Excessive G-CSF provokes neutrophilia in the bone marrow and these apoptosis-resistant neutrophils along with Ly6Chigh inflammatory monocytes (IM) invade the brainstem in massive numbers inflicting damage that culminates in fatal HSV encephalitis (HSE). G-CSF also suppresses IL-10 production by regulatory CD4 T cells resulting in an inability to regulate IM. Bottom panel: WT mice. IFNγ produced by WT T cells suppresses G-CSF production via increased SOCS3 expression thereby inducing neutrophil apoptosis and protecting WT mice from fatal HSE. Cells involved in this mechanism are shown in the key. Red arrows: inhibitory, green arrows: stimulatory; text in gray and dashed arrows indicate reduced or absent effectors. (TIF) [file ppat.1006822.s010.tif]
